# Neuropilin-2–expressing breast cancer cells mitigate radiation-induced oxidative stress through nitric oxide signaling

**DOI:** 10.1172/JCI181368

**Published:** 2024-10-01

**Authors:** Ayush Kumar, Hira Lal Goel, Christi A. Silva, Tao Wang, Yansong Geng, Mengdie Wang, Shivam Goel, Kai Hu, Rui Li, Lihua J. Zhu, Jennifer L. Clark, Lindsay M. Ferreira, Michael A. Brehm, Thomas J. FitzGerald, Arthur M. Mercurio

**Affiliations:** 1 Department of Molecular, Cell and Cancer Biology,; 2 Department of Radiation Oncology,; 3 Department of Pathology, and; 4 Department of Molecular Medicine, University of Massachusetts Chan Medical School, Worcester, Massachusetts, USA.

**Keywords:** Cell biology, Oncology, Breast cancer, Nitric oxide, Radiation therapy

## Abstract

The high rate of recurrence after radiation therapy in triple-negative breast cancer (TNBC) indicates that novel approaches and targets are needed to enhance radiosensitivity. Here, we report that neuropilin-2 (*NRP2*), a receptor for vascular endothelial growth factor (*VEGF*) that is enriched on subpopulations of TNBC cells with stem cell properties, is an effective therapeutic target for sensitizing TNBC to radiotherapy. Specifically, VEGF/NRP2 signaling induces nitric oxide synthase 2 (*NOS2*) transcription by a mechanism dependent on Gli1. NRP2-expressing tumor cells serve as a hub to produce nitric oxide (NO), an autocrine and paracrine signaling metabolite, which promotes cysteine-nitrosylation of Kelch-like ECH-associated protein 1 (*KEAP1*) and, consequently, nuclear factor erythroid 2-related factor 2–mediated (*NFE2L2*-mediated) transcription of antioxidant response genes. Inhibiting VEGF binding to NRP2, using a humanized mAb, results in NFE2L2 degradation via KEAP1, rendering cell lines and organoids vulnerable to irradiation. Importantly, treatment of patient-derived xenografts with the NRP2 mAb and radiation resulted in significant tumor necrosis and regression compared with radiation alone. Together, these findings reveal a targetable mechanism of radioresistance, and they support the use of NRP2 mAb as an effective radiosensitizer in TNBC.

## Abstract 2

We identified a pathway utilized by a subset of triple-negative breast cancer cells that is targetable using a humanized, function-blocking antibody to enhance the effects of radiotherapy.

## Graphical abstract

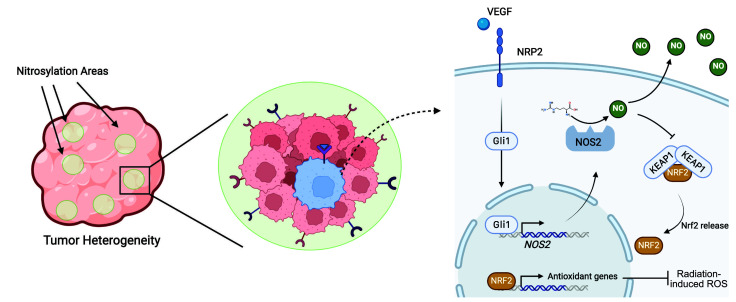

## Introduction

Triple-negative breast cancer (TNBC) is highly aggressive and is associated with a poor prognosis compared with other breast cancer subtypes (1, 2). Fortunately, several reports have shown that TNBC patients who attain a pathological complete response (pCR) after neoadjuvant chemotherapy have improved survival (3). As a result, there has been a greater emphasis on developing first-line treatments that can effectively achieve this histological criterion. However, with current neoadjuvant chemotherapy, many TNBC patients are diagnosed with residual disease, thus challenging clinicians with developing locoregional control strategies to improve long-term outcomes (4, 5). To this end, radiation therapy continues to be a recommended treatment modality as advances in artificial intelligence and imaging systems provide personalized treatment interventions that achieve optimal treatment delivery while minimizing side effects (6). Although radiotherapy substantially decreases recurrence rates and improves survival in a majority of breast cancer patients (7, 8), patients with TNBC do not have the same therapeutic response to radiotherapy compared with those with other breast cancer subtypes (9). Unfortunately, there are very few radiosensitizing agents available for this cohort of patients. Clinical trials in this field have assessed DNA-damage repair inhibitors and immune checkpoint blockers, but the results have been modest with significant variability in the response among patients (10, 11). Therefore, to improve treatment outcomes after radiation therapy, tumor-intrinsic features that are characteristic of TNBC need to be identified and assessed for their contribution to radioresistance.

The challenge associated with identifying biomarkers of radioresistance is the extensive intratumoral heterogeneity of TNBC and the diverse biological roles of its clonal populations. Emerging evidence indicates that cancer stem cells (CSCs), a subpopulation of cells capable of regulating intratumor heterogeneity, are highly enriched in TNBC compared with other subtypes (12, 13). Moreover, CSCs are unique in their ability to adapt to various stresses and modulate the tumor microenvironment to sustain protumorigenic functions (14, 15). Several studies have demonstrated an enrichment of CSCs within tumors after irradiation because of their ability to counteract the ROS generated through the hydrolysis of water (16–18). Radiosensitizers that target this function of CSCs could enhance the therapeutic effect of radiotherapy on tumor cells while sparing normal tissues. In this context, our lab has pioneered efforts demonstrating that vascular endothelial growth factor (*VEGF*) signaling via neuropilin-2 (*NRP2*) in tumor cells sustains CSC properties (19, 20). NRP2 is a single-pass transmembrane protein that is highly expressed in aggressive cancers, especially TNBC. Biological processes including tumor initiation, migration, and epithelial-to-mesenchymal transition are dependent on VEGF binding with NRP2 (19–21). However, an aspect of VEGF/NRP2 signaling that has not been investigated is the buffering of oxidative stress, which is critical for CSC function and has the potential to be leveraged to overcome radioresistance. Here, we establish this function of VEGF/NRP2 signaling, which involves S-nitrosylation of Kelch-like ECH-associated protein 1 (*KEAP1*) and, consequently, nuclear factor erythroid 2-related factor 2–mediated (*NFE2L2*-mediated) transcription of antioxidant response genes. Moreover, we demonstrate that this mechanism can be targeted using humanized mAbs (22) to enhance radiosensitivity in TNBC.

## Results

### Inhibition of VEGF/NRP2 enhances the radiosensitivity of TNBC.

**Figure 1:**
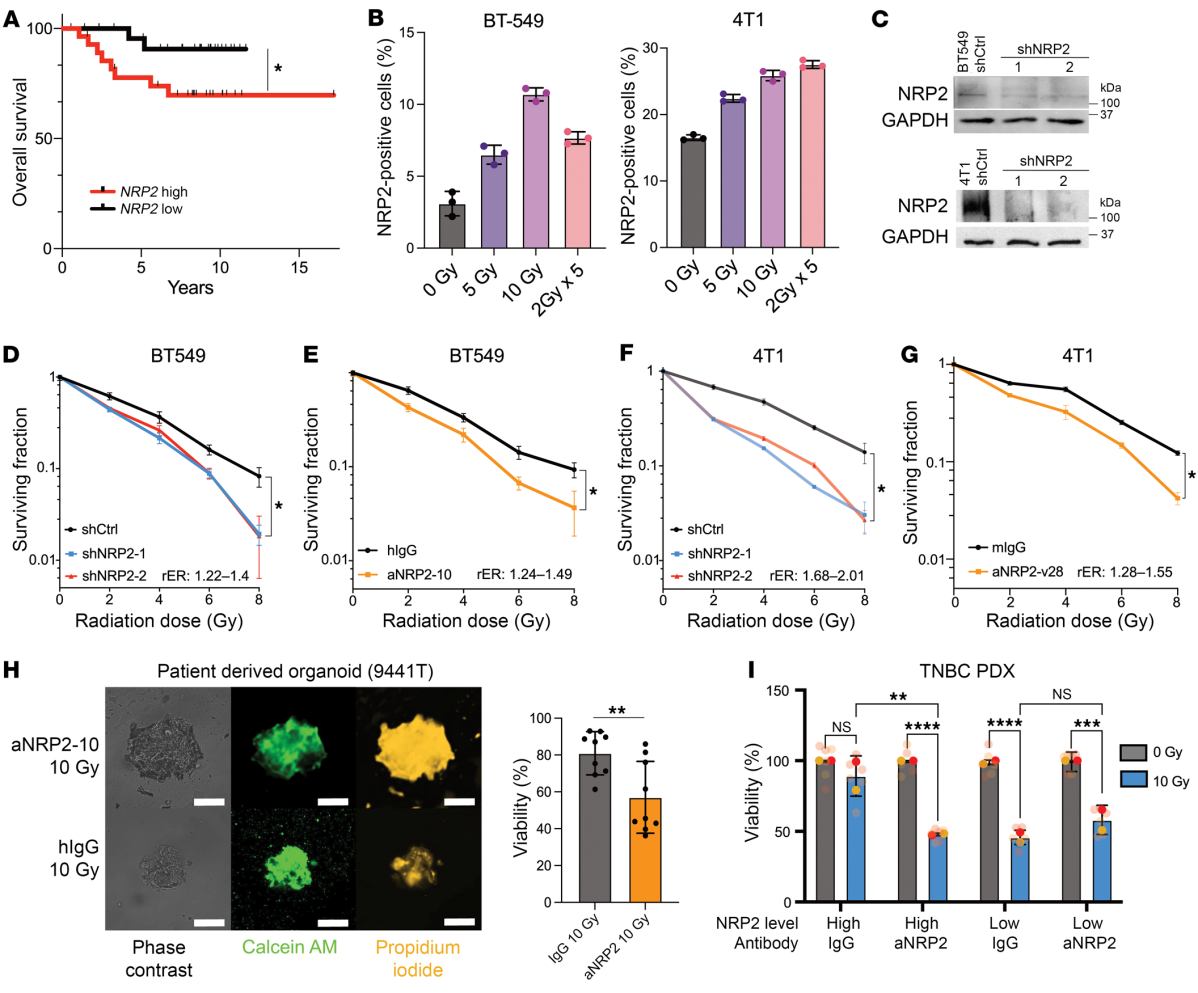
NRP2 expression modulates radiation sensitivity of TNBC models. (**A**) A Kaplan-Meier curve of overall survival for TNBC patients given radiotherapy and segregated based on median *NRP2* mRNA expression from GEO GSE199633 (*n* = 55). Gehan-Breslow-Wilcoxon test with *P < 0.05. (**B**) The TNBC cell lines indicated were given a radiation dose of 0, 5, 10 Gy, or 2 Gy × 5 and the percentage of cells with NRP2 surface expression was quantified by flow cytometry (*n* = 3). (**C**) Validation of NRP2 knockdown in BT549 and 4T1 cells transfected with shRNAs (shNRP2-1, shNRP2-2) compared with the cells transfected with a control (shCtrl) by immunoblotting. (**D**) Clonogenic assay of BT549 shCtrl, shNRP2-1, and shNRP2-2 cells after irradiation (0–8 Gy; *n* = 2, representative image). (**E**) Clonogenic assay of BT549 parental cells treated with either hIgG or aNRP2-10 and irradiated (0–8 Gy; *n* = 2, representative image). (**F**) Clonogenic assay of 4T1 shCtrl, shNRP2-1, and shNRP2-2 cells that had been irradiated (0–8 Gy; *n* = 2, representative image). (**G**) Clonogenic assay of 4T1 parental cells treated with either hIgG or aNRP2-28 and irradiated (0–8 Gy; *n* = 2, representative image).*P < 0.05. (**H**) CALYPSO-based analysis of organoid viability after treatment with either hIgG or aNRP2-10 and radiation (10 Gy). Calcein AM is a marker of live cells, and propidium iodide is a marker for dead cells. Scale bars: 100 μm. The bar graph shows the viability measurement for 10 organoids in each condition 48 hours after irradiation. **P < 0.01. (**I**) Viability of a PDX sorted for NRP2^hi^ and NPR2^lo^ expression and then treated with either aNRP2-10 or hIgG prior to irradiation (0 Gy or 10 Gy) was assessed 48 hours after irradiation (*n* = 2). Data are presented as means ± SD (**B**–**I**). Statistical analysis was performed using 2-tailed Student’s *t* test (**H**) or 2-way ANOVA multiple comparisons (**D**–**G** and **I**). **P < 0.01; ***P < 0.001; ****P < 0.0001.

First, we analyzed the gene -expression profile from a study that investigated the role of single-dose radiotherapy on normal human mammary and TNBC primary cells (23). We focused on genes encoding surface proteins that had increased expression upon radiation in TNBC, but did not change in normal mammary cells. There were 22 genes that met these criteria and could be strong candidates for targeted therapy with mAbs (Supplemental Table 1; supplemental material available online with this article; https://doi.org/10.1172/JCI181368DS1). One of these genes was *NRP2*, which is correlated with an unfavorable prognosis in breast cancer, especially in TNBC patients that receive radiotherapy (Figure 1A). We hypothesized that radiotherapy enriches for NPR2-expressing cancer cells within tumors; thus, we screened several TNBC-derived cell lines and observed a positive correlation between radiation dosage and NRP2 cell-surface expression (Figure 1B and Supplemental Figure 1, A and B). To evaluate whether *NRP2* expression is a critical mediator of survival after irradiation, we used RNA interference to reduce NRP2 protein expression in BT549, a human TNBC cell line, and 4T1, a mouse TNBC cell line (Figure 1C), and assessed apoptosis by annexin V binding, after a radiation dose of 4 Gy (Supplemental Figure 1C). Within 24 hours, there was a marked increase in the number of annexin V–positive cells among the NRP2-knockdown cells compared with the control cells. We then evaluated the radiosensitivity of these cells with clonogenic assays. Decreasing *NRP2* expression caused a significant increase in the radiosensitivity of BT549 cells compared with control cells (Figure 1D). We validated that this resistance is dependent on VEGF binding to NRP2 by using mAbs that block this binding (aNRP2-10 for human and aNRP2-28 for mouse cells) (22). Treatment of BT549 cells with aNRP2-10 increased the radiosensitivity enhancement ratio (rER) compared with IgG-treated cells (Figure 1E). We repeated these experiments with 4T1 cells and observed similar results (Figure 1, F and G). These data indicate that the VEGF/NRP2 axis provides a critical advantage in counteracting the cytotoxic effects of radiotherapy.

To evaluate the translational relevance of this data, we first assessed the conventional radiotherapy regimens used in the clinic for TNBC patients. The standard of care is fractionated irradiation of the local tumor site by delivering a total dose of 50 Gy over 25 fractions (24). We developed an in vitro model of radioresistance that mimics this standard-of-care treatment using BT549 cells and assessed the impact of resistance on NRP2 expression. The radioresistant cells (BT549-RR) had a significantly higher percentage of cells with NRP2 surface expression compared with control cells and were more resistant to radiotherapy (Supplemental Figure 1, D and E). Treatment of BT549-RR cells with aNRP2-10 sensitized them to radiation compared with control IgG (Figure 1F). We also developed radioresistant 4T1 cells (4T1-RR) using the same strategy and observed a similar increase in NRP2 surface expression and increased sensitivity to radiation when treated with aNRP2-28 (Supplemental Figure 1G).

In addition to cell lines, we used TNBC patient-derived organoids (PDOs) and patient-derived xenografts (PDXs) to assess the effectiveness of aNRP2-10 to enhance radiosensitivity. We assessed viability of the PDOs using CALYPSO (25, 26) and the Cell-Titer Glo luminescence assay. CALYPSO involves measuring the ratio of the intensity of viable cells (Calcein AM) to the sum of the intensity from the dead cells (propidium iodide) and viable cells using immunofluorescence (27). We observed that aNRP2-10 treatment sensitized PDOs to radiation (Figure 1H and Supplemental Figure 1H). After sorting a PDX based on NRP2 surface expression (Supplemental Figure 1I), we observed that the NRP2^lo^ population of tumor cells was significantly more sensitive to radiotherapy compared with the NRP2^hi^ population. However, the NRP2^hi^ population had increased sensitivity to radiation when pretreated with aNRP2-10 (Figure 1I).

### NRP2-expressing cells are a hub for nitric oxide signaling.

**Figure 2:**
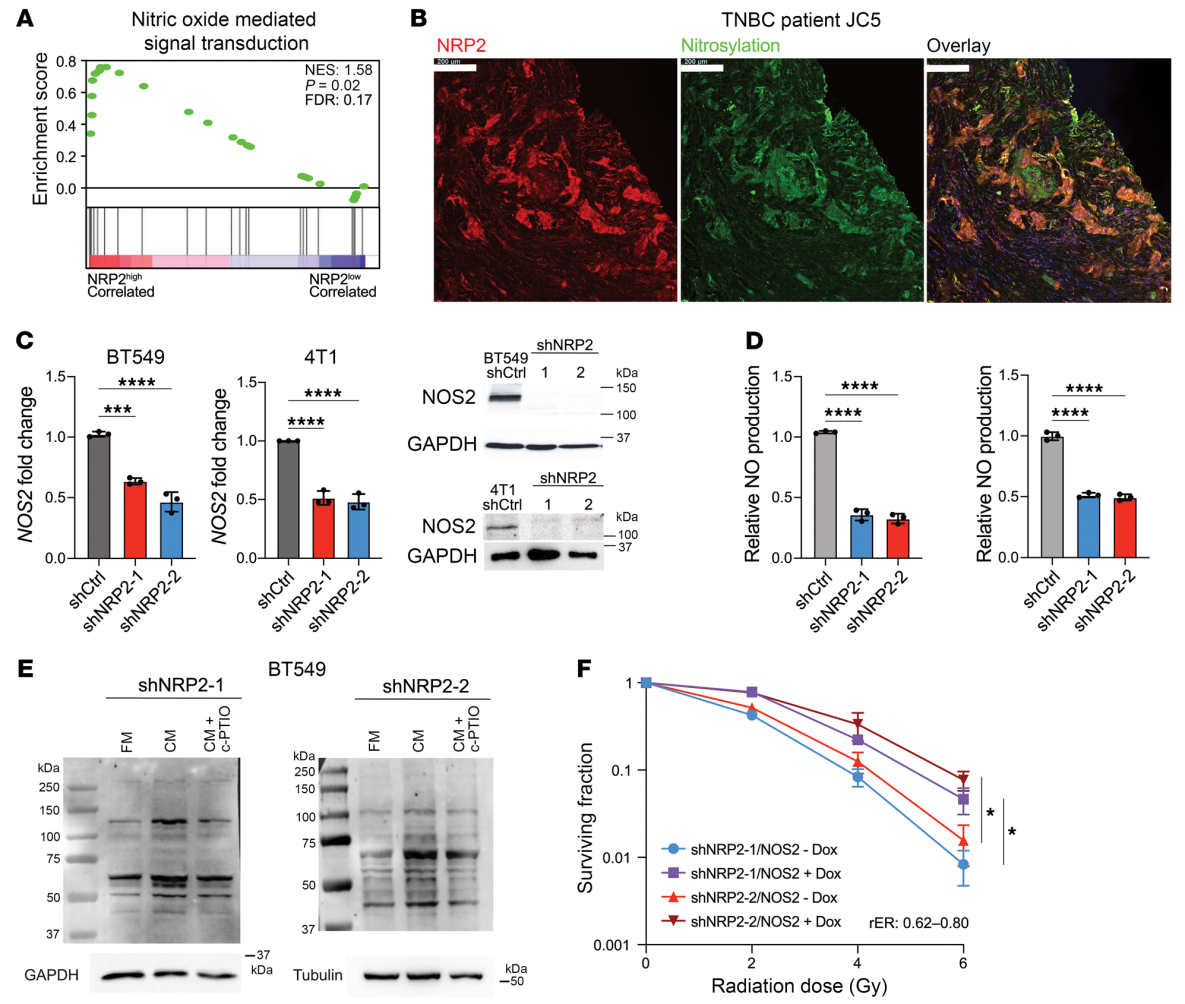
NRP2-expressing cells provide a hub for NO production. (**A**) The enrichment score associated with the nitric oxide–mediated signal transduction gene set from gene ontology biological pathways (GOBP). (**B**) Representative IHC images of a TNBC patient tumor immunostained with antibodies against NRP2, nitrotyrosine, and DAPI. Scale bars: 200 μm. (**C**) *NOS2* mRNA and protein expression in control and shNRP2 cells were quantified by qPCR and immunoblotting (*n* = 3). ***P < 0.001; ****P < 0.0001. (**D**) NO production in control and shNRP2 was estimated based on the Nitrite Assay Kit (*n* = 3). ****P < 0.0001. (**E**) Immunoblots of protein nitrotyrosine obtained from BT549 NRP2 knockdown cells given either control full media (FM), conditioned medium from NRP2^hi^ cells (CM), or c-PTIO (50 μM) that had been added to conditioned media from NRP2^hi^ cells (CM + c-PTIO). The conditioned media for the latter conditions was added to the NRP2 -knockdown cells 6 times over the course of 24 hours (*n* = 3, representative image). (**F**) Clonogenic assay of BT549 cells in which *NRP2* had been knocked down using 2 shRNAs and then transfected with t*NOS2* with and without doxycycline and irradiated (0–6 Gy; *n* = 2, representative image). *P < 0.05 Data are presented as means ± SD (**C**, **D** and **F**). Statistical analysis was performed using 1-way ANOVA multiple comparisons (**C** and **D**) or 2-way ANOVA multiple comparisons (**F**).

To understand the mechanism by which *NRP2* promotes radioresistance, we analyzed the transcriptomic differences between NRP2^hi^ and NRP2^lo^ populations of BT549 cells. The upregulation of nitric oxide synthase 2 (*NOS2*) (Supplemental Figure 2A) along with the enrichment of the gene set for nitric oxide–mediated (NO-mediated) signal transduction (Figure 2A), a unique bioactive messenger that is capable of initiating radioresistance (28, 29), were of particular interest to us. Subsequently, we assessed the expression and localization of nitrotyrosine, a surrogate marker of NO concentration, and NRP2 by fluorescence immunohistochemistry in TNBC specimens (Figure 2B and Supplemental Figure 2B). Although we observed significant intratumor heterogeneity in the localization of nitrotyrosine and NRP2, the high Pearson’s colocalization coefficient (Supplemental Figure 2C) demonstrated that there is a strong linear relationship between the 2 markers. Interestingly, the Mander’s colocalization coefficient, a measure of the fractional overlap of a probe with a second probe, for nitrotyrosine was much lower than the Mander’s coefficient for NRP2 (Supplemental Figure 2D). Given the well-documented capacity of NO as an intercellular messenger (30, 31), we postulate that NRP2-expressing cells provide a hub for nitrosylation by regulating *NOS2* expression.

We then evaluated the ability of VEGF/NRP2 to modulate *NOS2* expression. Downregulating *NRP2* expression reduced *NOS2* mRNA and protein expression in both BT549 and 4T1 cells (Figure 2C), validating our RNA-Seq data. To substantiate a causal role for VEGF in regulating *NOS2* expression, we inhibited the expression of *VEGF-C*, the predominant ligand for NRP2, with siRNA and observed diminished *NOS2* transcript levels (Supplemental Figure 2E). Moreover, disrupting the binding of VEGF-C with NRP2 using aNRP2-10 induced a significant reduction in *NOS2* transcript levels in BT549 and BT549-RR cells (Supplemental Figure 2F). As expected, the reduction in *NOS2* in NRP2 knockdown cells resulted in a 50% decrease in nitrite levels, a metabolite and surrogate measure of NO (Figure 2D). To assess the intercellular diffusion capability of NO, we collected the conditioned medium from NRP2^hi^ cells and added DMSO or c-PTIO, a NO scavenger, to the cell culture dish of NRP2 knockdown cells. We observed a significant increase in the nitrotyrosine content of the proteome for the conditioned medium group that was abrogated when incubated with c-PTIO (Figure 2E).

Next, we evaluated the functional relevance of the NRP2 nitrosylation hub in the context of radioresistance. Breast cancer patients that had been given radiotherapy were selected from The Cancer Genome Atlas (TCGA) and separated based on their expression of *NRP2* and *NOS2* mRNA. A Kaplan-Meier analysis demonstrated that patients with higher than median expression of both *NRP2* and *NOS2* had a shorter disease-free survival time compared with the group with low expression of the 2 genes (Supplemental Figure 2G). To verify that the NRP2/*NOS2* axis has a functional role in radioresistance, we inhibited NOS2 activity in BT549 cells using a chemical inhibitor, 1400W, and observed a significant decrease in the surviving fraction of cells over a wide range of radiation doses (Supplemental Figure 2H). We repeated this experiment by inhibiting *NOS2* expression with shRNAs, which increased the rER (Supplemental Figure 2I). Subsequently, we used a tetracycline-inducible *NOS2* plasmid (t*NOS2*) to increase *NOS2* expression in the NRP2-knockdown cells, which rescued the radioresistance phenotype (Figure 2F and Supplemental Figure 2J). Moreover, the conditioned medium from NRP2^hi^ cells, which is rich in NO, protected the NRP2-knockdown cells from radiation. This rescue of viability was nullified by the addition of c-PTIO (Supplemental Figure 2K). Importantly, we did not observe a change in radiosensitivity for the BT549 cells with t*NOS2* when treated with aNRP2-10 (Supplemental Figure 2L), indicating the necessity of *NOS2* repression upon aNRP2-10 treatment for modulating radiosensitivity.

### NRP2 induces NOS2 expression to mitigate radiation-induced ROS.

**Figure 3:**
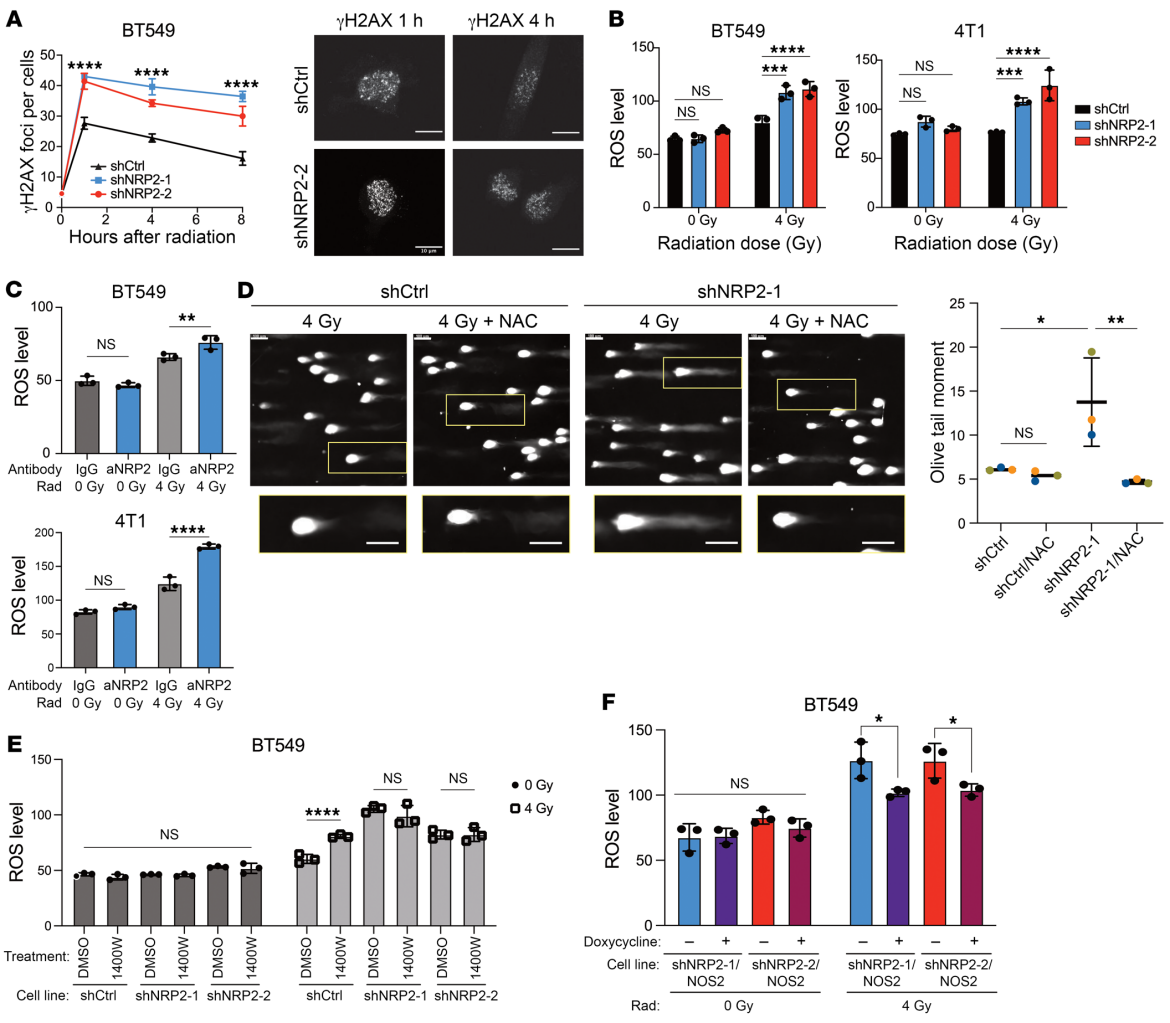
VEGF/NRP2 mitigates radiation-induced ROS via *NOS2*. (**A**) γ-H2AX foci in BT549 control and NRP2-knockdown cells were quantified by immunofluorescence at the time points indicated after 4 Gy irradiation (*n* = 3). Representative images of the foci at the respective time points and conditions. Scale bars: 10 μm. ****P < 0.0001. (**B**) ROS levels in BT549 and 4T1 shCtrl and shNPR2 cells were measured 4 hours after a 4 Gy radiation dose (*n* = 3). ***P < 0.001; ****P < 0.0001. (**C**) ROS levels in BT549 and 4T1 cells that had been pretreated with either IgG or aNRP2 for 24 hours were measured 4 hours after 4 Gy irradiation (*n* = 3). **P < 0.01; ****P < 0.0001.(**D**) DNA damage was quantified by the olive tail moment using the alkaline comet assay in BT549 shCtrl and BT549 shNRP2-1 cells 4 hours after 4 Gy irradiation, with or without NAC treatment 2 hours prior to radiation (*n* = 3). Scale bars: 100 μm. *P < 0.05; **P < 0.01. (**E**) The impact of NOS2 inhibition with 1400W (50 μM) on ROS levels in BT549 shCtrl and shNRP2 cells 4 hours after 4 Gy irradiation (*n* = 3). ****P < 0.0001. (**F**) ROS levels were measured 4 hours after 4 Gy radiation in NRP2-knockdown cells transfected with t*NOS2* with and without doxycycline (*n* = 3). *P < 0.05. Data are presented as means ± SD (**A**–**F**). Statistical analysis was performed using 2-tailed Student’s t test (**F**), 1-way ANOVA multiple comparisons (**D**), and 2-way ANOVA multiple comparisons (**A**–**C** and **E**).

In pursuit of the mechanism responsible for radioresistance driven by the nitrosylation capacity of NRP2-expressing cells, we evaluated the 2 key factors that mitigate radiation-induced cytotoxicity: DNA damage repair and oxidative stress management. A reliable metric for DNA damage repair is tracking the decay of γ H2AX foci over time after irradiation. Thus, we calculated the average number of foci in BT549 control and NRP2-knockdown cells over the course of 8 hours (Figure 3A). Although the rate of decay of foci for the 3 groups did not change significantly, we did observe a marked increase in the number of foci at all time points in the NRP2-knockdown cells compared with control cells. To substantiate this finding, we assessed whether the NRP2-knockdown cells had higher levels of ROS, which indirectly cause accumulated DNA damage within the cells. Using H2DCFDA, we saw a significant increase in radiation-induced ROS levels in the NRP2-knockdown cells compared with the control in BT549 and 4T1 cells (Figure 3B). Furthermore, treating BT549 and 4T1 parental cell lines with their respective NRP2 function-blocking antibodies increased ROS levels after irradiation (Figure 3C). However, this response was not evident when treating PDOs with aNRP2-14, which blocks binding of semaphorin 3F to NRP2 (Supplemental Figure 3A). We observed that increased ROS induced marked changes in DNA damage. Specifically, we pretreated cells with N-acetylcysteine (NAC), a strong antioxidant, for 2 hours prior to irradiation and assessed DNA damage using an alkaline comet assay. We observed longer tails in the NRP2-knockdown cells compared with the control cells. Moreover, NAC mitigated the DNA damage in the NRP2-knockdown cells but had no significant effect on the control cells (Figure 3D). Therefore, VEGF/NRP2 can minimize ROS accumulation induced by radiation and mitigate its associated DNA damage. To determine whether the buffering of ROS by VEGF/NRP2 is dependent on NOS2 activity, we treated BT549 cells with 1400W, which significantly increased ROS levels after irradiation only in the control cells and not in NRP2-knockdown cells (Figure 3E). Furthermore, the transient expression of *NOS2* in the NRP2-knockdown cells decreased ROS levels after irradiation compared with the control NRP2-knockdown cells that had intrinsically low levels of NO production (Figure 3F). These results indicate that the DNA damage driven by radiation-induced oxidative stress is mitigated by the NO generated by the NRP2/*NOS2* axis.

### VEGF/NRP2 regulates NOS2 transcription via Gli1.

**Figure 4:**
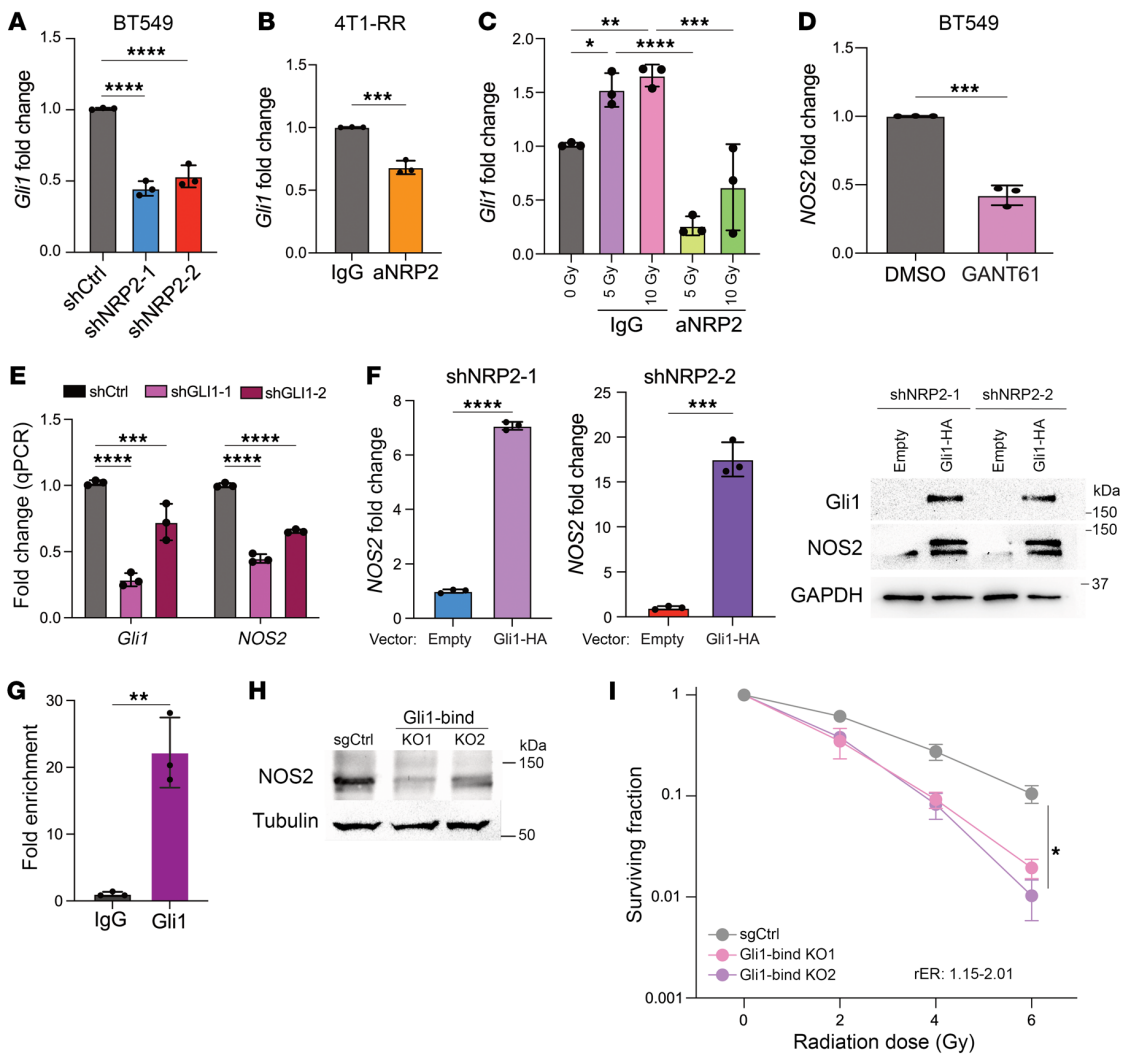
*NOS2* transcription is dependent on Gli1. We evaluated the *Gli1* mRNA expression in (**A**) BT549 shCtrl and shNRP2 cells (*n* = 3), (**B**) 4T1-RR cells that had been treated with either IgG or aNRP2-10 for 24 hours (*n* = 3), and (**C**) BT549 cells given a combined treatment of radiation (0, 5, and 10 Gy) with antibody for 24 hours (*n* = 3). *P < 0.05; **P < 0.01; ***P < 0.001; ****P < 0.0001. (**D**) *NOS2* mRNA expression was quantified in BT549 cells that had been treated with either DMSO or GANT61 (10 μM) for 24 hours (*n* = 3). ***P < 0.001. (**E**) *Gli1* and *NOS2* mRNA expression was quantified in BT549 shCtrl and shGli1 cells (*n* = 3). ***P < 0.001; ****P < 0.0001. (**F**) *NOS2* mRNA expression in BT549 shNRP2 cells that had been transfected with either empty vector or a *Gli1*-HA construct (*n* = 3). The immunoblot shows the protein expression of NOS2, Gli1, and GAPDH in the same cells. ***P < 0.001; ****P < 0.0001. (**G**) Binding of Gli1 on the *NOS2* promoter was analyzed using ChIP-qPCR in BT549 cells (*n* = 2, representative image). **P < 0.01. (**H**) NOS2 expression of CRISPR-generated mutations of the Gli1-binding site (Gli1-bind KO1 and KO2) compared with control (*n* = 3). (**I**) Clonogenic assay of control (sgCtrl), Gli1-bind KO1, and Gli1-bind KO2 cells that had been irradiated (0–6 Gy; *n* = 2, representative image). *P < 0.05 Data are presented as means ± SD (**A**–**G**, and **I**). Statistical analysis was performed using 2-tailed Student’s *t* test (**B**, **D**, and **F**), 1-way ANOVA multiple comparisons (**A**, **C**, and **E**), or 2-way ANOVA multiple comparisons (**I**).

Based on our previous report that *Gli1* expression is a downstream signaling event induced by VEGF/NRP2 (21), we confirmed that *Gli1* expression is dependent on VEGF binding to NRP2, as evidenced by its decreased expression in NRP2 knockdown BT549 compared with control cells (Figure 4A). We observed a decrease in *Gli1* expression in parental BT549 cells, BT549-RR, and 4T1-RR cells treated with NRP2 function blocking antibody (Figure 4B and Supplemental Figure 3B). Furthermore, radiation increased the expression of *Gli1*, but it was decreased in the presence of aNRP2-10 (Figure 4C). Given the stark changes in *Gli1* expression, we investigated whether *NOS2* is a Gli1 target gene. To test this hypothesis, we treated cells with GANT61, a Gli1 inhibitor, and observed a significant decrease in *NOS2* mRNA expression (Figure 4D). To substantiate these results, we used 2 shRNAs to knockdown *Gli1* expression, which also reduced *NOS2* mRNA levels (Figure 4E). Conversely, expression of a *Gli1*-HA construct in NRP2-knockdown cells increased *NOS2* expression (Figure 4F). *Gli1* is a direct transcriptional target of Hedgehog signaling, which is an integral part of several human malignancies (32); therefore, we looked to identify the impact of Gli2 and Gli3 on *NOS2* expression. Diminished expression of either *Gli2* or *Gli3* did not impact the expression of *NOS2* (Supplemental Figure 3C). Based on these results, we sought to identify potential binding sites for Gli1 near the *NOS2* promoter. We utilized a published Gli1 ChiP-Seq dataset (33) that identified a Gli1-binding site 2.5 kb upstream from the *NOS2* transcription start site (Supplemental Figure 3D). Subsequently, we performed ChIP-qPCR to validate binding of Gli1 to the promoter region of *NOS2* (Figure 4G). To establish that this structural element is a key driver of Gli1-induced *NOS2* transcription, we used paired single-guide RNAs (sgRNAs) to delete the specific regulatory element defined in Figure 4G using CRISPR/Cas9. After single-cell cloning, we selected 2 heterozygous knockouts of the Gli1-binding region, which were selected based on PCR amplification of the Gli1-binding region and gel electrophoresis (Supplemental Figure 3E). These clones had significantly lower expression of *NOS2* mRNA and protein compared with the control CRISPR/Cas9-treated cells (Figure 4H and Supplemental Figure 3F). Furthermore, these cells had decreased survival fraction after radiation compared with the control cells (Figure 4I). These data reveal a mechanism of *NOS2* expression that is dependent on Gli1.

### NFE2L2 activity is dependent on NRP2-induced NOS2 expression.

**Figure 5:**
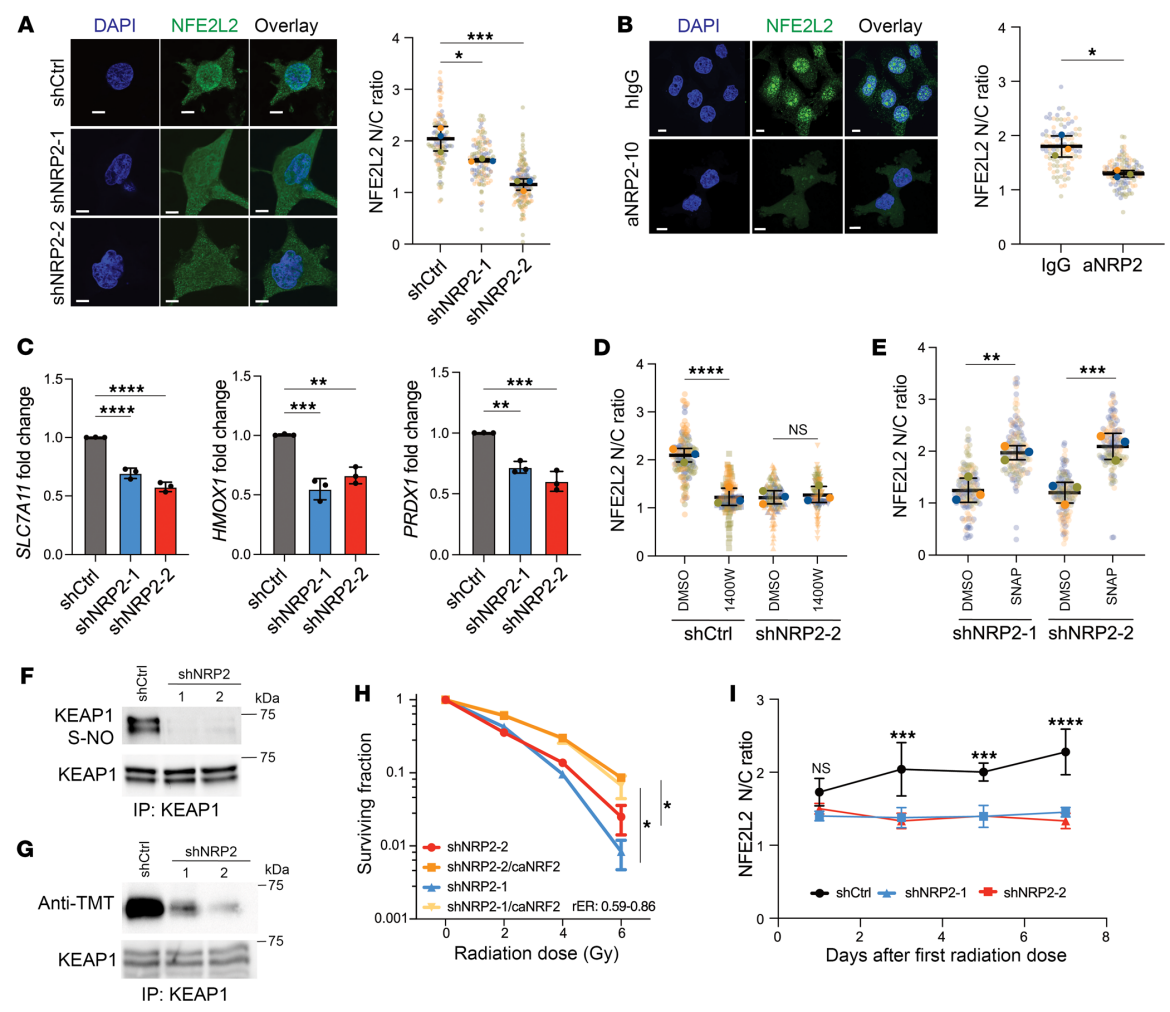
VEGF/NRP2 promotes S-nitrosylation of KEAP1 to activate NFE2L2. (**A**) Immunofluorescence images of DAPI and NFE2L2 staining in BT549 control and NRP2-knockdown cells with a calculation of the nuclear to cytoplasmic (N/C) ratio of NFE2L2 localization (*n* = 3). Scale bars: 10 μm. *P < 0.05; ***P < 0.001. (**B**) Immunofluorescence images of DAPI and NFE2L2 staining in BT549 cells treated with either IgG or aNRP2 for 24 hours with a calculation of the nuclear to cytoplasmic ratio of NFE2L2 (*n* = 3). Scale bars: 10 μm. *P < 0.05. (**C**) Expression of NFE2L2 target genes (*SLC7A11*, *HMOX1*, and *PRDX1*) in NRP2-knockdown cells was quantified by qPCR (*n* = 3).**P < 0.01; ***P < 0.001; ****P < 0.0001. (**D**) Control and shNRP2-2 BT549 cells were treated with 1400W (50 μM) for 24 hours, and NFE2L2 activation was assessed by its nuclear-to-cytoplasmic ratio based on immunofluorescence (*n* = 3). ****P < 0.0001. (**E**) NRP2-depleted BT549 cells were treated with either DMSO or the NO donor SNAP (50 μM) for 24 hours, and NFE2L2 localization was assessed by immunofluorescence (*n* = 3). **P < 0.01; ***P < 0.001. KEAP1 S-nitrosylation was detected by (**F**) biotin switch assay and (**G**) iodoTMT assay in control and NRP2-knocked down cells with immunoprecipitated KEAP1 used as a control. (**H**) Clonogenic assay of BT549 NRP2 knockdown cells engineered to express ca*NFE2L2* or empty vector and irradiated (0–6 Gy; *n* = 2, representative image). *P < 0.05. (**I**) NFE2L2 nuclear/cytoplasmic ratio assessed by IF of control and NRP2-knockdown cells after 4 Gy irradiation every day starting from day 0 until day 5 (*n* = 3). ***P < 0.001; ****P < 0.0001. Data are presented as means ± SD (**A**–**E**, **H**, and **I**). Statistical analysis was performed using 2-tailed Student’s *t* test (**B**), 1-way ANOVA multiple comparisons (**A**, **C**–**E**, and **J**), or 2-way ANOVA multiple comparisons (**H** and **I**).

After establishing the importance of NRP2/*NOS2* in mitigating oxidative stress and promoting radioresistance, we sought to identify downstream pathways that would be responsible for this phenotype by analyzing transcriptomic differences between the 4T1 and 4T1-RR cells. Differential gene expression analysis revealed that *NOS2* mRNA expression was increased in the radioresistant cells, substantiating our results in Figure 2 linking *NOS2* to radioresistance. We noted that NFE2L2/KEAP1 signaling was one of the positively enriched gene sets (Supplemental Figure 4A). NFE2L2 is a key regulator of antioxidant response elements to mitigate ROS levels in cancer cells and has been reported to be modulated by NO (34, 35). We hypothesized that VEGF/NRP2 promotes NFE2L2 localization to the nucleus by inducing S-nitrosylation of KEAP1, a component of the cullin 3-based ubiquitin ligase (36). NRP2-knockdown cells exhibited a reduction in the nuclear localization of NFE2L2 compared with control cells based on immunofluorescence staining (Figure 5A). We observed similar results with BT549 cells treated with aNRP2-10 compared with IgG (Figure 5B). NFE2L2 stabilization by NRP2 downstream signaling was confirmed by the decrease in NFE2L2 target genes (*SLC7A11*, *HMOX1*, and *PRDX1*) in the NRP2-knockdown cells compared with control cells (Figure 5C). To demonstrate that the activity of NFE2L2 is *NOS2* dependent, we diminished *NOS2* expression using shRNAs, which induced a significant decrease in antioxidant genes (Supplemental Figure 4B). Moreover, treating cells with a *NOS2* inhibitor reduced NFE2L2 nuclear localization in control cells, but it had no significant effect on the NRP2-knockdown cells (Figure 5D).

To assess whether NRP2-expressing cells are hubs of NO signaling, we first treated control BT549 cells with the NO scavenger c-PTIO and observed a downregulation in the expression of *SLC7A11* and *HMOX1* transcripts (Supplemental Figure 4C). Furthermore, the conditioned medium from the control cells increased the expression of these genes in the NRP2-knockdown cells, which were decreased by adding c-PTIO (Supplemental Figure 4D). Adding SNAP, a NO donor, to NRP2-knockdown cells increased the NFE2L2 nuclear localization (Figure 5E). The NRP2-knockdown cells that were transfected with t*NOS2* demonstrated an increase in expression of NFE2L2 target genes (Supplemental Figure 4E). To validate that the expression of NFE2L2 target genes in NRP2-expressing cells is dependent on its NO production and subsequent S-nitrosylation of KEAP1, we used the biotin switch and iodoTMT switch assays. Both assays involve adding an additional moiety to SNO, either biotin or iodoTMT, followed by immunoprecipitation of KEAP1 and immunoblotting for streptavidin or anti-TMT, respectively. There was a significant reduction in KEAP1 S-nitrosylation in the NRP2-knockdown BT549 cells (Figure 5, F and G). Thus, we postulated that rescuing NFE2L2 stabilization in the NRP2-knockdown cells should increase radioresistance. For this purpose, we used a constitutively active *NFE2L2* (ca*NFE2L2*) plasmid, which does not have a KEAP1-binding domain, and transfected it into the NRP2-knockdown cells. The expression of ca*NFE2L2* increased radioresistance (Figure 5H and Supplemental Figure 4F). Furthermore, we knocked down *KEAP1* in the NRP2-knockdown cells and observed an increase in the surviving fraction after irradiation compared with the controls (Supplemental Figure 4, G and H).

An important aspect of NFE2L2 activation that needed to be addressed is its dependence on NRP2 in the context of radiation. Several reports have mentioned that NFE2L2 activation can be driven by radiation, especially after several days of fractionated radiation (37, 38). Nevertheless, we found that fractionated radiation was only able to induce NFE2L2 activation in the control cells, whereas the NRP2 knockdown cells did not have this response (Figure 5I). This result indicates that tumors rely on NRP2-expressing cells to promote NFE2L2 activation during radiotherapy and, consequently, promote resistance.

### Single dose or conventional fractionated radiotherapy combined with VEGF/NRP2 inhibition delays tumor growth.

**Figure 6:**
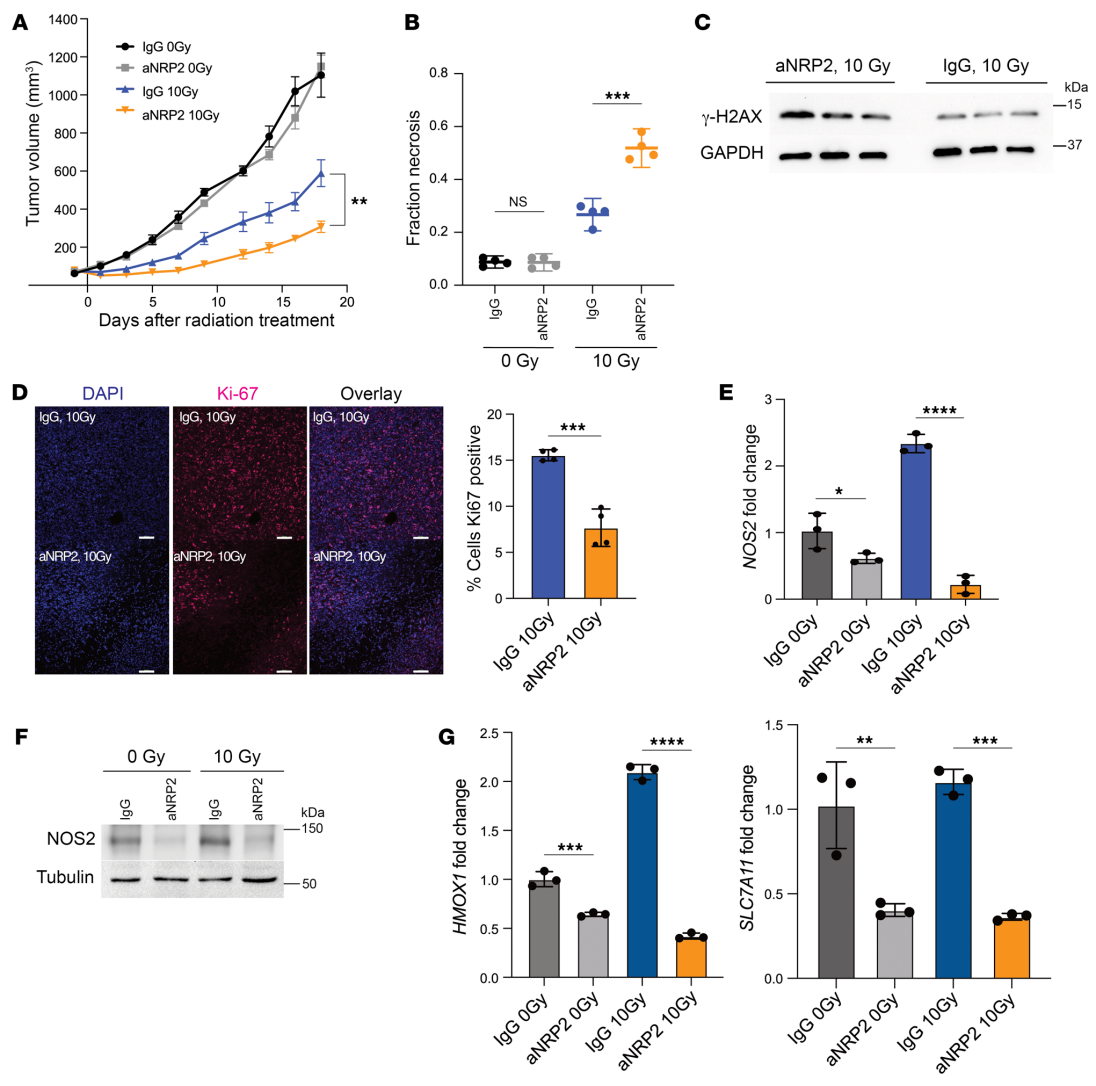
Single-dose radiotherapy with aNRP2 mitigates tumor growth. (**A**) 4T1 cells (5 × 10^5^) were injected into the mammary fat pads of BALB/c mice. Once the tumor volume reached approximately 100 mm^3^, the mice were divided into 4 groups of 7 mice each (mouse IgG, 0 Gy; mouse IgG, 10 Gy; aNRP2, 0 Gy; aNRP2, 10 Gy). The mice were given i.p. injections of the specified antibody (25mg/kg) every 48 hours starting 1 day prior to irradiation for 2 weeks. Tumors were extracted on day 18 and were used for histological and molecular profiling. **P < 0.01. (**B**) Necrotic areas of tissue sections of tumors were measured after H&E staining by finding the fraction of the area that is necrotic compared with the area of the tumor (*n* = 4). ***P < 0.001. (**C**) Immunoblot showing γ-H2AX protein levels in irradiated tumors that had been treated with either mIgG or aNRP2-28. (**D**) Cell proliferation in tumors from each treatment group was measured by Ki-67 immunofluorescence and quantified as a percentage of cells that were positive (*n* = 4). Scale bars: 100 μm. ***P < 0.001. (**E**) *NOS2* mRNA and (**F**) NOS2 protein levels were quantified for each treatment group using qPCR and immunoblotting, respectively (*n* = 3). ****P < 0.0001. (**G**) mRNA expression of NFE2L2 target genes (*SLC7A11* and *HMOX1*) was measured for each treatment group using qPCR (*n* = 3). **P < 0.01; ***P < 0.001; ****P < 0.0001. Data are presented as means ± SEM (**A**) and mean ± SD (**B**, **D**, **E**, and **G**). Statistical analysis was performed using 2-tailed Student’s *t* test (**D**), 1-way ANOVA multiple comparisons (**B**, **E**, and **G**), or 2-way ANOVA multiple comparisons (**A**).

**Figure 7:**
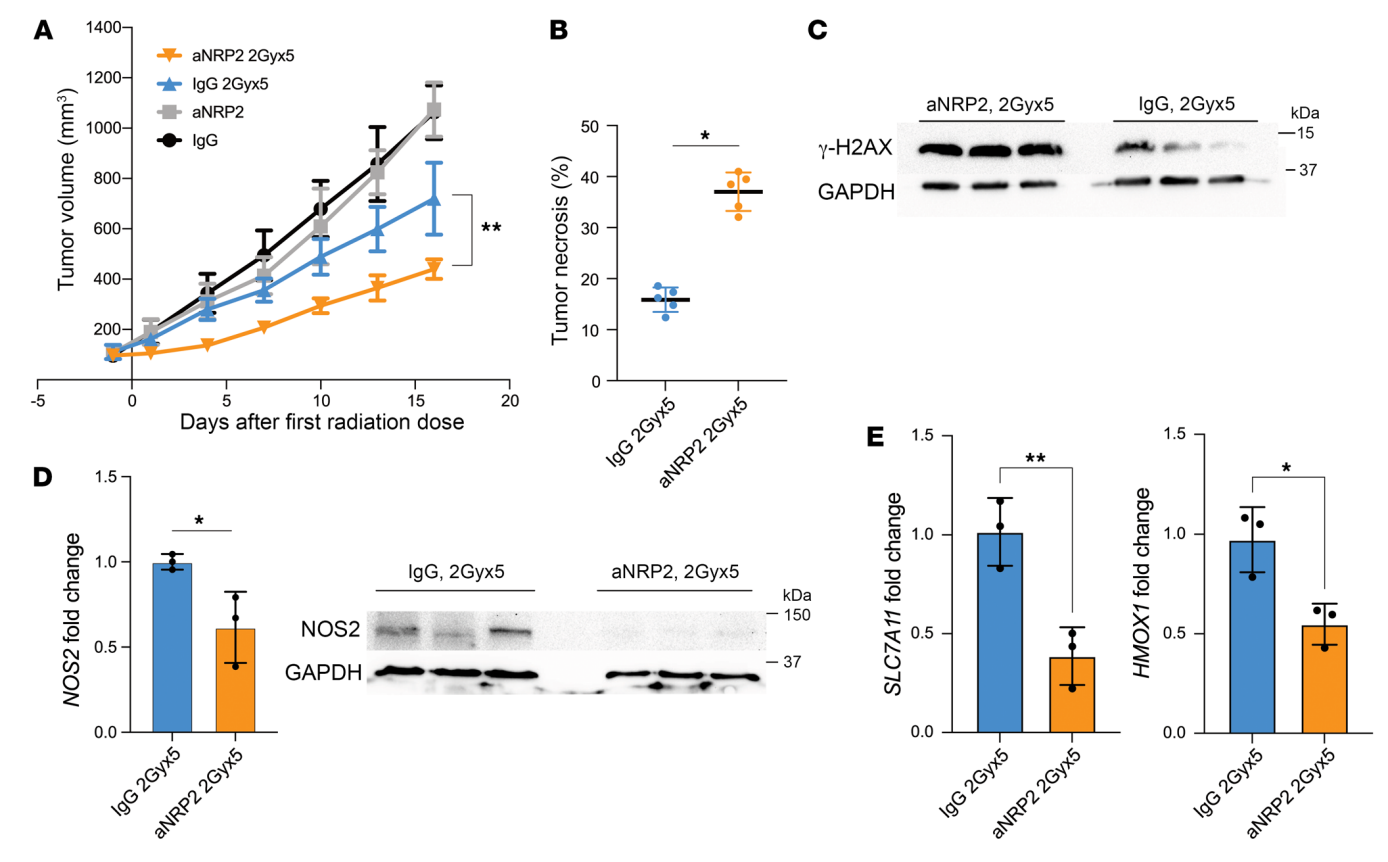
Conventional fractionation with aNRP2 mitigates tumor growth. (**A**) 4T1 cells (5 × 10^5^) were injected into the mammary fat pads of BALB/c mice. Once the tumor volume reached approximately 100 mm^3^, the mice were divided into 4 groups of 5 mice each (mouse IgG, 2Gyx5; mouse IgG, 2Gyx5; aNRP2, 2Gyx5; aNRP2, 2Gyx5). The mice were given i.p. injections of the specified antibody (25 mg/kg) every 48 hours starting 1 day prior to irradiation for 2 weeks. Tumor volumes were measured with calipers every 2 days and are shown as means ± SEM. Tumors were extracted on day 16 and were used for histological and molecular profiling. **P < 0.01. (**B**) Necrotic areas of tissue sections of tumors were measured after H&E staining by finding the fraction of the area that is necrotic compared with the area of the tumor (*n* = 5).*P < 0.05. (**C**) Immunoblot showing γ-H2AX protein levels in irradiated tumors that had been treated with either mIgG or aNRP2-28. (**D**) *NOS2* mRNA and protein levels were quantified for each treatment group using qPCR and immunoblotting (*n* = 3). *P < 0.05. (**E**) mRNA expression of NFE2L2 target genes (*SLC7A11* and *HMOX1*) was measured for each treatment group using qPCR (*n* = 3). *P < 0.05; **P < 0.01. Data are presented as means ± SD (**B**, **D**, and **E**). Statistical analysis was performed using 2-tailed Student’s *t* test (**B**, **D**, and **E**) or 2-way ANOVA multiple comparisons (**A**).

To investigate the potential of VEGF/NRP2 inhibition to enhance radiosensitivity in vivo, we first employed a syngeneic xenograft model using murine 4T1 TNBC cells and single-dose radiotherapy. A combination of a single-fraction 10 Gy irradiation dose with aNRP2-28 treatment significantly suppressed tumor growth compared with either treatment alone (Figure 6A). Also, combination treatment increased necrosis in the tumor compared with other treatment options (Figure 6B and Supplemental Figure 5A). Using retention of γH2AX as a reliable marker of radiosensitivity (39, 40), we observed that the VEGF/NRP2-function blocking antibody increased γH2AX expression compared with IgG control after irradiation (Figure 6C). To evaluate proliferation capacity of the tumors, we analyzed Ki-67 expression and quantified the number of mitotic cells. The tumors treated with aNRP2-28 had a significant reduction in the percentage of cells positive for Ki-67 (Figure 6D) and fewer mitotic cells (Supplemental Figure 5B), indicating that the VEGF/NRP2 function blocking antibody also limits tumor proliferation after radiotherapy. Importantly, we substantiated our previous results that VEGF/NRP2 inhibition represses expression of *NOS2* (Figure 6, E and F) and NFE2L2 target genes (Figure 6G). The irradiated tumors had higher expression of *NOS2* and NFE2L2 target genes compared with the controls, and treating with aNRP2-28 mitigated the expression of these pathways. We then proceeded to analyze the effects of conventional fractionated radiotherapy (2Gyx5 or 5 consecutive days with 2 Gy each day) with aNRP2-28. Similar to the single-dose radiotherapy experiment, the combination of VEGF/NRP2 inhibition with conventional fractionation radiotherapy mitigated the growth potential of the tumor (Figure 7A), increased necrosis within the tumor (Figure 7B), and increased the retention of γH2AX (Figure 7C) compared with the IgG with conventional fractionated radiation group. Moreover, the cotreatment of aNRP2-28 and radiation decreased expression of *NOS2* (Figure 7D) and NFE2L2 target genes (Figure 7E) compared with the conventionally fractionated group alone. With regards to the role of aNRP2 and safety, there were no significant changes in mouse weight (Supplemental Figure 5, C and D) or histological changes to the normal breast parenchyma (Supplemental Table 2).

### Hypofractionated radiotherapy with aNRP2 promotes tumor regression.

**Figure 8:**
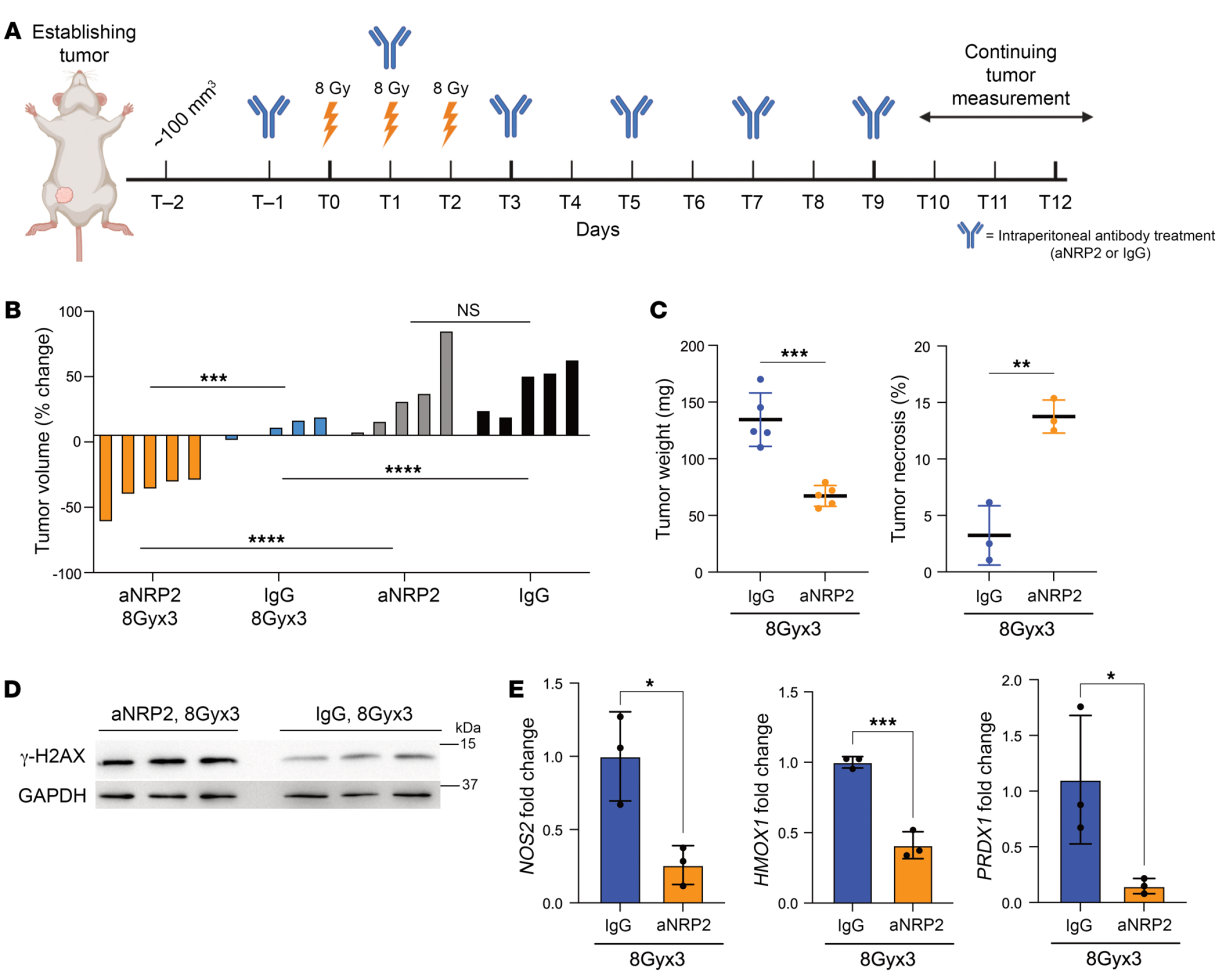
Hypofractionated radiation with aNRP2 promoted tumor regression in PDX model. (**A**) Schematic of the fractionated radiation and antibody-treatment schedules. (**B**) Tumor volumes in mice that had been implanted orthotopically with a human TNBC PDX in NSG mice. The mice were divided into 4 groups of 5 mice each. When tumors reached approximately 125–150 mm^3^, the mice were treated with either IgG (10 mg/kg), aNRP2-10 (10 mg/kg), IgG 8Gyx3, or aNRP2-10 8Gyx3. Antibody treatments were given as i.p. injections. The waterfall plot shows the percentage change in growth of the tumor from day –1 to day 15 for each individual mouse. Molecular and histological analysis of the tumors were done on day 15 after the first radiation dose. ***P < 0.001; ****P < 0.0001. (**C**) The tumor weights from the radiation-treated groups (*n* = 5), and percentage of tumor necrosis based on H&E section of the tumors from the radiation-treated groups (*n* = 3). **P < 0.01; ***P < 0.001. (**D**) Immunoblot of γ-H2AX from 3 mice in each of the fractionated radiation-treated groups. (**E**) *NOS2*, HMOX1, and *PRDX1* mRNA expression was quantified by qPCR from 3 mice in each of the radiation-treated groups. *P < 0.05; ***P < 0.001. Data are presented as means ± SD (**C** and **E**). Statistical analysis was performed using 2-tailed Student’s *t* test (**C** and **E**) or 1-way ANOVA multiple comparisons (**B**).

Advancements in irradiation technology and several clinical trials have promoted the use of hypofractionated radiotherapy in early stage, low-risk breast cancer. The standard of care generally requires 50 Gy in 25 fractions, whereas hypofractionation reduces the number of fractions to 5–15 fractions, with higher doses of radiation per fractionation (41–44). Although moderate hypofractionation and ultrahypofractionation are not clinically approved strategies for TNBC treatment in the US, we wanted to investigate the use of this radiation regimen with VEGF/NRP2 inhibition to evaluate its therapeutic efficacy in a preclinical setting. In this experiment, we used an aggressive PDX model of TNBC, HCI028 (45). When tumors reached the appropriate size, we began radiation and antibody treatments as outlined in Figure 8A. Neither fractionated radiation nor aNRP2-10 alone had an impact on tumor growth, which is consistent with known insensitivity of TNBC to radiation. However, combined treatment resulted in significant tumor regression (Figure 8B), reduced tumor weight, and increased necrosis (Figure 8C). We utilized the same experimental approach with the 4T1 xenograft model, which also resulted in tumor regression, reduced tumor weight, and increased necrosis in the combined treatment group compared with the fractionated radiation alone (Supplemental Figure 6, A and B). Also, higher levels of γH2AX were detected in tumors from the combined treatment group compared with the other groups (Figure 8D and Supplemental Figure 6C). In support of our previous findings in this study, we observed a significant reduction in the expression of *NOS2* and NFE2L2 target genes in tumors from the combined treatment group compared with radiation alone (Figure 8E and Supplemental Figure 6D). Finally, the cotreatment of aNRP2 with hypofractionated radiation had no effect on mouse weight compared with the hypofractionated group alone in either the PDX or 4T1 models (Supplemental Figure 6, E and F).

## Discussion

This study uncovered a signaling pathway in breast cancer that can be exploited to sensitize tumors to radiation therapy. Specifically, a subpopulation of cells in TNBC with stem cell properties that is dependent on VEGF/NRP2 signaling functions as a hub for the localized production of NO that promotes the S-nitrosylation of effector proteins such as KEAP1 that enable the expression of NFE2L2-mediated antioxidant genes. Consequently, tumor cells can buffer the accumulation of radiation-induced ROS and mitigate its downstream effects, including DNA damage and cell death. We were able to hinder the nitrosylation capacity of these cells using a function-blocking NRP2 mAb, which sensitizes TNBC organoids and orthotopically implanted tumors, including PDXs, to radiation therapy. Given that the investigation of tumor-intrinsic factors in TNBC that are targetable and responsible for radioresistance is still in its infancy, our study provides a significant advance in this area.

Our data reinforce the importance of CSCs in radioresistance, and they establish a specific mechanism that sustains this resistance, i.e., VEGF/NRP2 signaling. This conclusion is supported by our finding that radiotherapy selects for the survival of tumor cells with high NRP2 surface expression and that inhibiting VEGF/NRP2 sensitizes this population to radiation. We infer from these data that a minority population of cells with stem cell properties exists in TNBC that is inherently radioresistant because of VEGF/NRP2 signaling. The possibility exists, however, that radiotherapy induces the surface expression of NRP2 in some cells based on the reports that NRP2 can localize in the cytoplasm in some tumor cells and that its trafficking can be regulated by external stimuli (46, 47). Our efforts to demonstrate this latter possibility, however, were inconclusive.

NO-mediated signaling, especially from *NOS2*, is an integral factor in driving the aggressive phenotype and therapy resistance seen in TNBC patients (48, 49). In fact, several reports have shown endogenous NO production relays compensatory mechanisms to mitigate the effects of radiotherapy (29, 50). The next frontier in NO biology is understanding its spatiotemporal control within the tumor microenvironment, and our work provides 2 crucial insights into its regulation. We report that NRP2-expressing cells serve as a hub for NO production, by enhancing *NOS2* transcription via Gli1, which creates local fields within the tumor that can protect the cells from radiation. It is worth noting that much of the literature regarding *NOS2* regulation in TNBC tumors has centered around cytokine signaling, hypoxia, and stress hormones (51–53). Our data provide a unique pathway of *NOS2* regulation independent of immune and hormonal factors that is specific to TNBC tumors.

Our findings on protein S-nitrosylation as a mechanism of radioresistance merit discussion. We investigated S-nitrosylation because it is the dominant mode by which NO signals and is a tightly regulated process that modulates several cellular functions (54, 55). We established that a key mechanism of NO-mediated radioresistance is the S-nitrosylation of KEAP1, which induces the expression of NFE2L2-antioxidant genes. Although some studies have characterized the importance of this pathway in mitigating ROS levels in cancer cells, they were based on the use of exogenous NO donors. We provide data that the endogenous production of NO by VEGF/NRP2 is sufficient to initiate S-nitrosylation of KEAP1 in TNBC. This posttranslational modification provides the initiation of antioxidant genes that can mitigate ROS accumulation after irradiation.

The translational impact of our study is evidenced by the ability of mAbs that block the binding of VEGF to NRP2 to sensitize TNBC to radiotherapy in several preclinical models. There are a myriad of radiation regimens used to treat breast cancer, and there is still active investigation regarding their safety and efficacy in TNBC. We investigated these approaches in our animal models using VEGF/NRP2 function-blocking mAbs and observed enhanced radiosensitivity that resulted in regression in tumor growth and increased necrosis using single-dose, conventional fractionation, and hypofractionated radiation. These results provide justification for the initiation of clinical trials using aNRP2-10, a humanized mAb, to enhance radiosensitivity. The feasibility of such trials is strengthened by studies that have shown that aNRP2-10 is highly specific for inhibiting the binding of VEGF to NRP2 and that it did not exhibit toxicity in animal models, including primates (22). In addition, aNRP2-10 has been manufactured for clinical use. It is also worth noting that bevacizumab, which has been used in clinical trials (56), does not block the binding of VEGF to NRP2 but to VEGF receptor tyrosine kinases (57).

We are aware of limitations to our study. Although aNRP2-10 has been evaluated in nonhuman primates and found not to have detectable toxicity, it has yet to be tested in humans, which will require a phase 1 clinical trial. Also, there are limited tools available to directly track the spatial distribution of NO in vivo, which precluded us from determining the impact of VEGF/NRP2 inhibition on the diffusion distance and half-life of NO in tumors.

In summary, the data demonstrate that NRP2-expressing cells utilize their role as nitrosylation hubs, which drives the expression of NFE2L2-dependent antioxidant genes to counteract radiation-induced oxidative damage. Current radiosensitizers undergoing clinical trials for TNBC treatment focus on immune checkpoint blockade (10, 11). However, the response to such radiosensitizers is dependent on stochastic features such as immune cell penetrance in the tumor, activation of antitumor immunity, and immune cell exhaustion. In contrast, aNRP2-10 should potentiate radiation therapy more consistently compared with these radiosensitizers, especially given the high expression of NRP2 in TNBC and the inherent need of antioxidant mechanisms for cancer cells to mitigate radiation-induced ROS.

## Methods

### Sex as a biological variable

The mice utilized in this study were female because human breast cancer largely affects female patients.

### Cell culture

The BT-549, BT-20, MDA-MB-468, and Hs578t human breast cancer cell lines and the 4T1 mouse breast cancer cell line were purchased from ATCC and were authenticated by the University of Arizona Genetic Core (UAGC). We developed radioresistant models of BT549 and 4T1 by giving a total of 50 Gy over the course of 8 weeks using the following treatment schedule: 2Gyx5, 4Gyx3, 6Gyx3, and 10Gyx1 (58). Before moving on to the next radiation dose, we waited for the cells to reach 70%–80% confluency in the plate.

### PDOs

The UMass Chan Tumor Bank (Worcester, Massachusetts, USA)collected biopsies from TNBC patient tumors that were deidentified before utilizing them for experiments. The PDO we utilized was labeled as T9441. The tumor tissue was digested with the Tissue Dissociation Kit (Miltenyi Biotech) and gentleMACS Dissociator. The dissociated tumor was embedded into reduced growth factor basement membrane extract (Cultrex).

### Reagents

Calcein AM was purchased from Cayman Chemical (14948). Propidium iodide was purchased from Thermo Fisher Scientific (P1304MP). Annexin V–FITC was purchased from Invitrogen (A13199). The NOS2 inhibitor, 1400W, was purchased from Abcam (ab120165). S-nitroso-*N*-acetyl-DL-penicillamine was purchased from MedChemExpress (HY-121526). Carboxy-PTIO was purchased from Caymen Chemical (81540). GANT61 was provided by Rune Toftgård (Karolinska Institutet, Solna, Sweden). The NRP2 antibodies (aNRP2-10 and aNRP2-28) and mouse IgG antibody were provided by aTyr Pharma. The human IgG antibody was purchased from SinoBiological (catalog HG4K). The following antibodies were used for immunoblotting: tubulin (Cell Signaling Technology, 3873), β-actin (Cell Signaling Technology, 3700S), GAPDH (14C10) (Cell Signaling Technology, 2118S), human NRP2 (aNRP2-36v2 obtained from aTyr; ref. 59), mouse NRP2 (R&D Systems, AF2215), human NOS2 (Cell Signaling Technology, 39898), mouse NOS2 (D6B6S) (Cell Signaling Technology, 13120), nitrotyrosine antibody (Santa Cruz Biotechnology, sc-32757), Gli1 (Cell Signaling Technology, 2553s), KEAP1 (D6B12) (Cell Signaling Technology, 8047s), and phospho-histone H2A.X (Ser139) (20E3) (Cell Signaling Technology, 9718s). The following Abs were used for flow cytometry: NRP2 (R&D Systems, AF2215) and anti-goat Alexa Fluor 488 (Invitrogen A-11055). For immunofluorescence, the following antibodies were used: NRF2 (D1Z9C) (Cell Signaling Technology, 12721s), phospho-histone H2A.X, Ki67 (BioLegend, 652401), NRP2 (abcam, ab234821), nitrotyrosine, anti-rabbit-FITC (Invitrogen, F-2765), anti-rat-AF647 (Invitrogen, A-48272), anti-mouse-AF488 (Invitrogen, A-11001), and anti-rabbit-AF647 (Invitrogen, A-21245).

### Constructs

The following lentiviral shRNAs were obtained from our core facility: human NRP2 (TRCN0000063309, TRCN0000063312), mouse NRP2 (TRCN0000063309 and TRCN0000063310), NOS2 (TRCN0000003764, TRCN0000003765), Gli1 (TRCN0000020484, TRCN0000020488), and KEAP1 (TRCN0000155340, TRCN0000158081). shCtrl vectors were pLKO scramble shRNAs (Addgene, 1864). Lentivirus packaging vectors were obtained from Addgene pMD2.G (12259 and psPAX2 12260). A lentiviral plasmid expressing t*NOS2* was obtained from Addgene (110800). A *Gli1*-HA tagged construct was provided by Martin Fernandez-Zapico (Mayo Clinic, Rochester, Minnesota, USA). A constitutively active *NFE2L2* plasmid was obtained from James Alvarez (Fred Hutchinson Cancer Center, Seattle, Washington, USA).

### Engineered cell lines

To generate lentivirus, the packaging plasmids were cotransfected with the lentiviral plasmids in human embryonic kidney-293T cells using lipofectamine 3000 (Thermo Fisher Scientific, catalog L3000008). The media was collected from these cells 48 hours after transfection and filtered through a 0.45 μm filter. The virus was added to the cells with polybrene and then made stable through selection with puromycin for 2 weeks.

To transiently knock down VEGF-C, we transfected siRNAs into cells using lipofectamine 3000 and analyzed the mRNA expression after 48 hours. The siRNAs used for this experiment were as follows: negative control DsiRNA, hs.Ri.VEGFC.13.1 CCAACCGAGAAUUUGAUG, and hs.Ri.VEGFC.13.2 CAACAACAAUUGGUAAAACUCACTG.

To overexpress *NOS2*, we prepared lentivirus for the t*NOS2* plasmid, infected the cells of interest, and made them stable with puromycin. To initiate *NOS2* expression, we added 10 μM doxycycline to the media and waited at least 24 hours before assessing changes in *NOS2* expression, setting up a clonogenic assay, or evaluating ROS levels after irradiation.

We used the following guides to delete the Gli1-binding region near the *NOS2* promoter: 5′-GTCTGTGATGCACACCACGC-3′, 5′-GCTGTGAGAAGGTAAACATG-3′. The following reagents were purchased from IDT: Alt-R CRISPR crRNA (2 nmol), CRISPR/Cas9 tracrRNA (catalog 1072532), and Cas9 nuclease (Alt-RTM S.p. Cas9 nuclease 3NLS, catalog 1074181); these were used to assemble the Cas9:crRNA:tracrRNA RNP complex. The RNP complexes were transfected in cells, one at a time, using the Nucleofector Device (Lonza Biologics) with the program X-005 and Nucleofector Kit V (Amaxa VCA-1003). Cells were cultured for 48 hours and then underwent single-cell sort.

For each clone, a portion of the cells were taken for DNA extraction. The region of the Gli1-binding region was amplified with PCR with the specified primers (5′-TGCTTGGTGTGGCATTCT-3′,5′-GCCGATATGGCATCCTGATTA-3′) using the KOD Hot Start DNA Polymerase Kit (Sigma Aldrich, 71086). After PCR amplification, 20 μL of product was mixed with 4 μL of 6× Agarose Gel Loading Dye (Boston BioProducts, BM-100G). The DNA products were separated with a 2% agarose gel with SYBR Safe DNA Gel Stain (Invitrogen, S33102) and then imaged using an EpiChemi 3 darkroom.

### Clonogenic assays

Cells were treated with the appropriate radiation dose, and a single-cell suspension was collected 24 hours later. A predetermined number of cells was added to each well and evenly distributed based on cell type and radiation dose. The media in the wells was replaced every 3 days. After 10–14 days, the plates were fixed with 4% paraformaldehyde for 15 minutes, washed 3 times with 1× PBS, and stained with 0.5% crystal violet in 80% methanol for 45 minutes. Colonies with more than 50 cells were counted. The plating efficiency was calculated as the number of colonies formed divided by the number of cells added to the well for the nonirradiated control. The surviving fraction was calculated based on the ratio of colonies in the treatment group to the number of colonies in the nonirradiated samples and the plating efficiency of the cell line. For antibody-treatment groups, the cells were pretreated 48 hours prior to receiving radiation at a concentration of 10 μg/mL and once during the plating of cells. The rER was calculated by measuring the area under the curve from the clonogenic assay of the control to the experimental group.

### Cell-survival assays

For the PDO, we utilized CALYPSO to assess viability of organoids. The organoids were given 10 days to grow after seeding to reach an appropriate size. The antibody treatments (10 μg/mL) were given 24 hours prior to a radiation dose of 10 Gy. After 72 hours, the organoids were stained with Calcein AM (8 μM) and propidium iodide (4 μM) in 0.1 mM CuSO4 for 30 minutes. Subsequently, the organoids were washed with 1× PBS 3 times for 5 minutes each time. The organoids were imaged with a Nikon confocal microscope with *Z*-stacks for each organoid. The cell viability was calculated based on the intensity of Calcein AM compared with the summation of intensities of Calcein AM and propidium iodide (PI). For both the PDO and PDX, we assessed viability in vitro using the CellTiter-Glo 3D reagent (Promega, G9681). The viability percentage was calculated based on the luminescence of the treated samples compared with the untreated.

### Flow cytometry

To assess surface expression of NRP2 in cell lines, we incubated 1 × 10^6^ cells in 100 μL of PBS with primary antibody at a concentration of 1:100 for 30 minutes on ice. The cells were washed with PBS and centrifuged for 3 minutes at 300*g*. This washing process was done 3 times. After the last wash, the cells were resuspended in 100 μL of PBS and incubated with secondary antibody at a concentration of 1:1,000. Apoptosis after irradiation was assessed using annexin V binding to the cell surface. The cells were collected and resuspended in annexin-binding buffer (10 mM HEPES, 140 mM NaCl, and 2.5 mM CaCl_2_, pH 7.4) and aliquoted in a glass tube to have 1 × 10^5^ cells in 100 μL. Then 5 μL of annexin V-FITC was added to the solution along with 3 μL of 100 μM solution of PI. The samples were incubated in the dark for 15 minutes. An additional 400 μL of the annexin-binding buffer was added to the solution and then placed on ice until analyzed on the flow cytometer (BD FACS Celesta).

### Biochemical assays

#### NO measurement.

The concentration of NO was determined using the Measure-IT High-Sensitivity Nitrite Assay Kit (Invitrogen M36051). We removed the supernatant from the cells and used the kit to determine the concentration and then we counted the number of cells to get the relative NO production per cell for each experimental group.

#### ROS quantification.

For the quantification of ROS levels, we used the fluorescent probe 2′,7′–dichlorofluorescin diacetate (H2DCFDA). For adherent cells, we added 5,000 cells from each condition into a well of a 96-well plate and waited 24 hours for attachment. For antibody-treated conditions, the antibody was added at the same time. Next, we treated the cells with a 4 Gy radiation dose and waited 4 hours before we stained the cells with 5 μM H2DCFDA for 30 minutes and washed with 1× PBS 3 times for 5 minutes each. The microplate was analyzed with the Promega GloMax plate reader.

#### Alkaline comet assay.

To assess the DNA damage of the cells and its dependence on radiation-induced ROS, we followed the following protocol (60). Cells were suspended in 2.5 × 10^4^ cells/100 μL of 0.7% low-melting point agarose. Two 40 μL drops were added to a precoated agarose slide, which was covered with a 20 × 20 mm coverslip and allowed to gel for 5 minutes. The slides were submerged in alkaline lysis buffer (1.2M NaCl, 100 mM Na_2_EDTA, 0.1% sodium laryl sarcosinate, and 0.26 M NaOH) overnight at 4°C. The slides were washed in rinse solution (0.03 M NaOH, 2 mM Na_2_EDTA) for 20 minutes. The slides were placed in an electrophoresis chamber with the same rinse solution under constant voltage of 22 V for 35 minutes at 4°C. The slides were stained in 2.5 μg/mL of propidium iodide for 20 minutes. Images were taken using the Nikon confocal microscope and analyzed using the OpenComet software in Fiji (61).

#### Immunoblotting.

Protein extraction was done by scraping cells on ice with RIPA buffer (BP-115DG, Boston Bioproducts) supplemented with protease and phosphatase inhibitors (Thermo, 78442). Subsequently, laemmli buffer (BP-111R, Boston Bioproducts) was added to each sample and the lysate was boiled and separated using SDS-PAGE. Immunoblotting primary antibodies were used at the following concentrations: NRP2, 1:1,000; human NOS2, 1:1,000; mouse NOS2, 1:1,000; tubulin and GAPDH, 1:2,000; γH2AX, 1:1,000; nitrotyrosine, 1:1,000; Gli1, 1:1,000; KEAP1, 1:1,000; streptavidin HRP, 1:1,000; and anti-TMT 1:1,000. The secondary antibodies conjugated with HRP were used at a concentration of 1:5,000. mRNA quantification was completed by first extracting RNA using the NucleoSpin RNA Kit (Macherey-Nagel 740955.50) and proceeding to cDNA synthesis (AZ-1996, Azura Genomics). The relative expression levels were quantified using the Azura View GreenFast qPCR Blue Mix LR Master Mix (AZ-2320, Azura Genomics). Experiments were performed in triplicate and normalized to *GAPDH*. All primers were obtained from the Harvard PrimerBank and are listed in Supplemental Table 3.

### RNA-Seq

RNA was extracted from the indicated cells using a QIAGEN RNeasy Micro Kit (74004) and sent to Quick Biology (BT549 NRP2^hi^ versus NRP2^lo^) or Novogene (4T1-Par vs 4T1-RR) for sequencing. The library for RNA-Seq was prepared according to KAPA Stranded mRNA Hyper Prep PolyA Selected Kit with 201–300 bp insert size (KAPA Biosystems) using 250 ng total RNAs as input. Final library quality and quantity was analyzed by Agilent Technologies 4200 station and Qubit 3.0 (Thermo Fisher Scientific). For the Qubit 3.0 Fluorometer (Thermo Fisher Scientific), the 150 bp paired-end reads were sequenced on Illumina HiseqX (Illumnia Inc.). Each sample had a sequencing depth of 20–30 million. RNA-Seq analysis was performed with OneStopRNAseq workflow (62). Paired-end reads were aligned to human primary genome hg38, with star_2.5.3a (63). Aligned exon fragments with mapping quality higher than 20 were counted toward gene expression with featureCounts_1.5.2 (64). Differential expression (DE) analysis was performed with DESeq2. Significant DE genes (DEGs) were filtered with the criteria FDR < 0.05.

### Irradiation

For in vitro assays, the cells would receive the expected radiation dose either on a cell culture dish or a 15 mL conical tube. The linear accelerator with a collimator was set to 0° and a field size of 30 × 30 cm^2^. A 5 cm thick gel pack was placed under the dish or tube followed by a 1 cm thick gel pack placed on top of it. The x-ray beams were set at a dose rate of 300 cGy/min.

For in vivo experiments, mice were given IP injections of ketamine/xylazine (100 mg/kg of ketamine and 10 mg/kg of xylazine). After the mouse was anesthetized, it was placed supine on a styrofoam board with its appendages taped down with surgical tape. The LINAC had an energy output of 6MeV at a dose rate of 300 cGy/min. The collimated beam irradiated a circular area with a 1 cm diameter, which directed the radiation dose to the site of the tumor. During irradiation, a tissue equivalent bolus was placed on the skin.

### Immunofluorescence microscopy

For cell imaging, cells were cultured on 35 mm glass-bottom dishes, washed with PBS, and fixed with 4% paraformaldehyde in PBS for 15 minutes at room temperature. Subsequently, cells were washed with PBS, put in blocking buffer (5% normal goat serum [Sigma-Aldrich, G9023], 0.3% Triton X-100 in PBS) for 1 hour, and then incubated with primary antibodies in antibody dilution buffer (1% BSA, 0.3% Triton X-100 in PBS) overnight at 4°C. Cells were washed with PBS 3 times followed by 1 hour incubation with secondary antibodies and DAPI (1:1,000, Invitrogen). The cells were mounted in a 0.1M *n*-propyl gallate, 90% (by volume) glycerol, and 10% PBS solution.

For formalin-fixed, paraffin-embedded tissue samples, 5 μm thick sections were mounted onto slides and baked overnight at 65°C. The slides were dewaxed by washing in xylene 3 times for 5 minutes each, followed by washes in 100%, 95%, 90%, 75%, and 50% ethanol for 3 minutes each. The slides were rehydrated in ddH_2_O and then added to antigen retrieval solution (EnVision FLEX Target Retrieval Solution High pH, 50×) (Dako, DM828) and heated at 97°C using a pressure cooker chamber for 20 minutes. Slides were then washed in washing buffer (0.1 M tris-HCl, 0.3 M NaCl, 0.1% Tween 20, and 7.7 mM NaN_3_ [pH 7.6] at 25°C) twice for 10 minutes each. The sections were then incubated in blocking buffer (5% normal goat serum, 0.1 M tris-HCl, and 0.15 M NaCl [pH 7.6] at 25°C) for 30 minutes. After blocking, 100 μL of the primary antibodies in antibody dilution buffer were added to the slide and kept in a light-tight humid box overnight at 4°C. The next day, the slides were washed 3 times with washing buffer. Next, the slides were incubated with secondary antibodies and DAPI in antibody dilution buffer for 1 hour. Slides were washed 3 times in washing buffer, then mounted using ProLong Diamond Antifade Mountant (Thermo Fisher Scientific, P36970) and stored in the dark overnight. The overlap between the nitrotyrosine and NRP2 fluorescence was quantified using 2 metrics: Pearson and Mander’s correlation coefficients, which were calculated by JaCOP (65).

To assess the number of γH2AX foci after irradiation, we used the difference of Gaussians approach. We duplicated the image and applied a Gaussian blur filter of σ 1 and σ 2 for each image. Then, we used the image calculator to subtract the σ 2 image from the σ 1 image. We then counted the number of foci for each nucleus.

To assess the nuclear localization of NFE2L2, we developed masks for the nuclear region and whole cell and quantified the total fluorescent intensity in the field.

### S-nitrosylation assays

To detect differences in S-nitrosylation of KEAP1, we used the biotin switch assay (Cayman, 10006518) and the iodoTMT switch assay (Thermo, 90105) with immunoblotting. Both kits had prepared reagents, which were used for the following steps. The cells from each condition were cultured and lysed to collect a protein sample and then we proceeded with KEAP1 immunoprecipitation. Individual samples were diluted to ensure the protein concentration and total volume were the same. Each solution was then incubated with blocking buffer (to block free thiols), reducing buffer (to selectively reduce nitrosylated cysteines), and labeling buffer, with washes in between each step. The labeling buffer would covalently attach S-nitrosothiols with maleimide-biotin or iodoTMT. Samples would be mixed with 6× reducing Laemmli SDS sample buffer, heated on a 95°C block, and separated by SDS-PAGE.

### ChiP

We used the ChIP-IT Express Chromatin Immunoprecipitation Kit (Active Motif). The following primers amplified the region of the *NOS2* promoter with a Gli1 peak: 5′-GAGGGAAAGGAGGAAAGGAAAG-3′, 5′-CTGGAAGCCTACAACTGCAT-3′.

### Animal studies

The tumor models used in this study included the orthoptic injection of 4T1 cells into the mammary fat pads of 6- to 8-week-old female, BALB/c mice from Charles River Laboratories and the orthotopic implantation of HCI-031 in the mammary fat pads of 6- to 8-week-old female, NSG mice (NOD.Cg-Prkdc*^scid^* Il2rg*^tm1Wjl^*/SzJNSG) purchased from Jackson Laboratory. HCI-031 was derived from the pleural effusion of a patient with metastatic TNBC (45). The details of each experiment are described in Figures 6, 7, and 8 and Supplemental Figure 6. All reagents were administered via IP injections. Tumor volume was measured by measuring length and width of the tumor using calipers and applying the following equation: ½ × length × width^2^. Once the tumor reached the specified endpoint, we euthanized the mice and harvested the tissue.

### Statistics

Two-tailed Student’s *t* test was used to compare between 2 groups, and more than 2 groups were compared using either 1-way ANOVA or 2-way ANOVA followed by Tukey’s multiple-comparison test. Kaplan-Meier analysis was completed using the Gehan-Breslow-Wilcoxon test. All statistical tests were carried out using GraphPad Prism, version 10.0, with a significance level set at *P* less than 0.05.

### Study approval

The experiments were approved by the University of Massachusetts IACUC (PROTO202100107).

### Data availability

The RNA-Seq data have been deposited in the NBCI’s Gene Expression Omnibus database (GEO GSE272955 [NRP2^hi^ versus NRP2^lo^]; GSE272692 [4T1RR versus 4T1Par]). Values for all data points in graphs are reported in the Supporting Data Values file. Raw immunoblot data are reported in the full unedited blot and gel images file.

## Author contributions

AK and HLG contributed to study design and analyzed data. AK, HLG, MW, and SG collected data for all experiments. KH, RL, and LJZ completed RNA-Seq analysis. TW, YG, and TJF provided support with radiation planning. AK, CAS, LMF, and MAB helped set up animal experiments. JLC performed histological analysis. AK and AMM wrote the manuscript.

## Supplementary Material

Supplemental data

Unedited blot and gel images

Supporting data values

## Version 1 10/01/2024

In-Press Preview

## Version 2 11/15/2024

Electronic publication

## Version 3 09/04/2025

Updated name of Christi A. Wisniewski to Christi A. Silva
