# Supplementary material for: Neuropilin-2–expressing breast cancer cells mitigate radiation-induced oxidative stress through nitric oxide signaling
Source: J Clin Invest. 2024 Oct 1;134(22):e181368. doi: 10.1172/JCI181368 (PMC11563673; doi:10.1172/JCI181368)
Supplement: Supplemental data [file jci-134-181368-s009.pdf]

Neuropilin-2 expressing breast cancer cells mitigate radiation-induced oxidative stress through nitric oxide signaling

Ayush Kumar[1], Hira Lal Goel[1], Christi A. Wisniewski[1], Tao Wang[2], Yansong Geng[2], Mengdie Wang[1], Shivam Goel[1], Kai Hu[1], Rui Li[1], Lihua Julie Zhu[1], Jennifer L Clark[3], Lindsay M Ferreira[4], Michael A Brehm[4], Thomas J FitzGerald[2], Arthur M Mercurio[1]

Departments of Molecular, Cell and Cancer Biology[1], Radiation Oncology[2], Pathology[3], and Molecular Medicine[4], University of Massachusetts Chan Medical School, Worcester MA.

Correspondence:

Arthur M. Mercurio, Ph.D.
Department of Molecular, Cell and Cancer Biology
UMass Medical School
LRB-428
364 Plantation Street
Worcester, MA 01605
phone: 508-856-8676
e-mail: arthur.mercurio@umassmed.edu

Conflict-of-interest statement: The authors have declared that no conflict of interest exists.

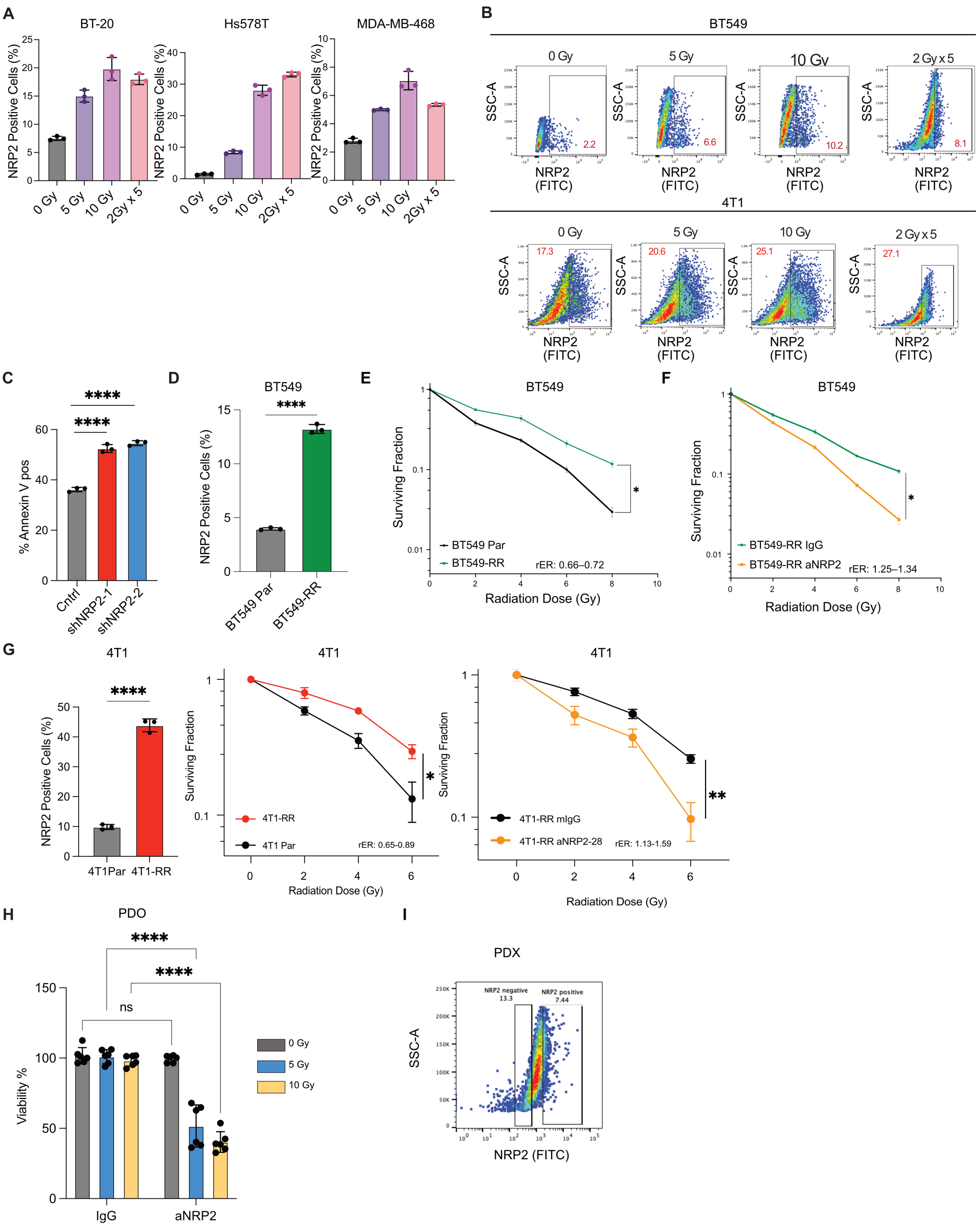
A
BT-20
Hs578T
MDA-MB-468
NRP2 Positive Cells (%)
0 Gy
5 Gy
10 Gy
2Gy x 5
B
BT549
0 Gy
5 Gy
10 Gy
2 Gy x 5
SSC-A
NRP2 (FITC)
2.2
6.6
10.2
8.1
4T1
17.3
20.6
25.1
27.1
C
% Annexin V pos
Cntrl
shNRP2-1
shNRP2-2
****
D
BT549
NRP2 Positive Cells (%)
BT549 Par
BT549-RR
E
BT549
Surviving Fraction
BT549 Par
BT549-RR
rER: 0.66–0.72
Radiation Dose (Gy)
F
BT549
BT549-RR IgG
BT549-RR aNRP2
rER: 1.25–1.34
G
4T1
NRP2 Positive Cells (%)
4T1Par
4T1-RR
4T1-RR
4T1 Par
rER: 0.65-0.89
4T1-RR mIgG
4T1-RR aNRP2-28
rER: 1.13-1.59
H
PDO
Viability %
ns
0 Gy
5 Gy
10 Gy
IgG
aNRP2
I
PDX
NRP2 negative
13.3
NRP2 positive
7.44
SSC-A
NRP2 (FITC)

Supplemental Figure 1: NRP2 expression modulates radiosensitivity (**A**) The TNBC cell lines indicated were given a radiation dose of 0, 5, 10Gy, or 2Gy x 5 and the percentage of cells with NRP2 surface expression was quantified by flow cytometry (n = 3). (B) The percentage of BT549 and 4T1 cells with NRP2 surface expression was quantified by flow cytometry 48h after treatment with either 0, 5, 10Gy, or 2Gyx5 radiation. **(C)** BT549 cells that had been transfected with either shCtrl or NRP2 shRNAs were irradiated (4Gy) and apoptosis was quantified by Annexin V flow cytometry (n = 3). (**D**) The percentage of BT549 parental vs radioresistant (RR) cells with NRP2 surface expression was quantified by flow cytometry (n = 3). (**E**) Clonogenic assay of BT549 and BT549-RR cells that had been irradiated (0-8 Gy; n = 2, representative image). (**F**) Clonogenic assay of BT549-RR cells treated with either hIgG or aNRP2-10 and irradiated (0-8 Gy; n = 2, representative image). **(G)** The percentage of 4T1 parental and 4T1-RR cells expressing NRP2 on their cell surface, clonogenic assay comparing radiation sensitivity of 4T1par and 4T1-RR, and clonogenic assay comparing radiation sensitivity of 4T1-RR when treated with aNRP2-28 or mIgG (n = 2, representative image). **(H)** A PDO (T9441) was treated with either aNRP2-10 vs IgG treatment at varying doses of irradiation (0,5, and 10Gy). Viability was assessed 48 hours after irradiation using the CellTiter-Glo luminescence assay (n=2, two independent experiments with three technical replicates). **(I)** Flow cytometry data of PDX tumor cells (HCI-031) and the gating strategy used to select the $NRP2^{high}$ and $NRP2^{low}$ populations of the PDX. Data are presented as the mean ± SD in **A, C**-**H**. Statistical analysis was performed using 2-tailed Student's t test in **D** and **G** (left), 1-way ANOVA multiple comparisons in **C** and **H**, or 2-way ANOVA multiple comparisons in **E**, **F**, and **G** (middle and right).

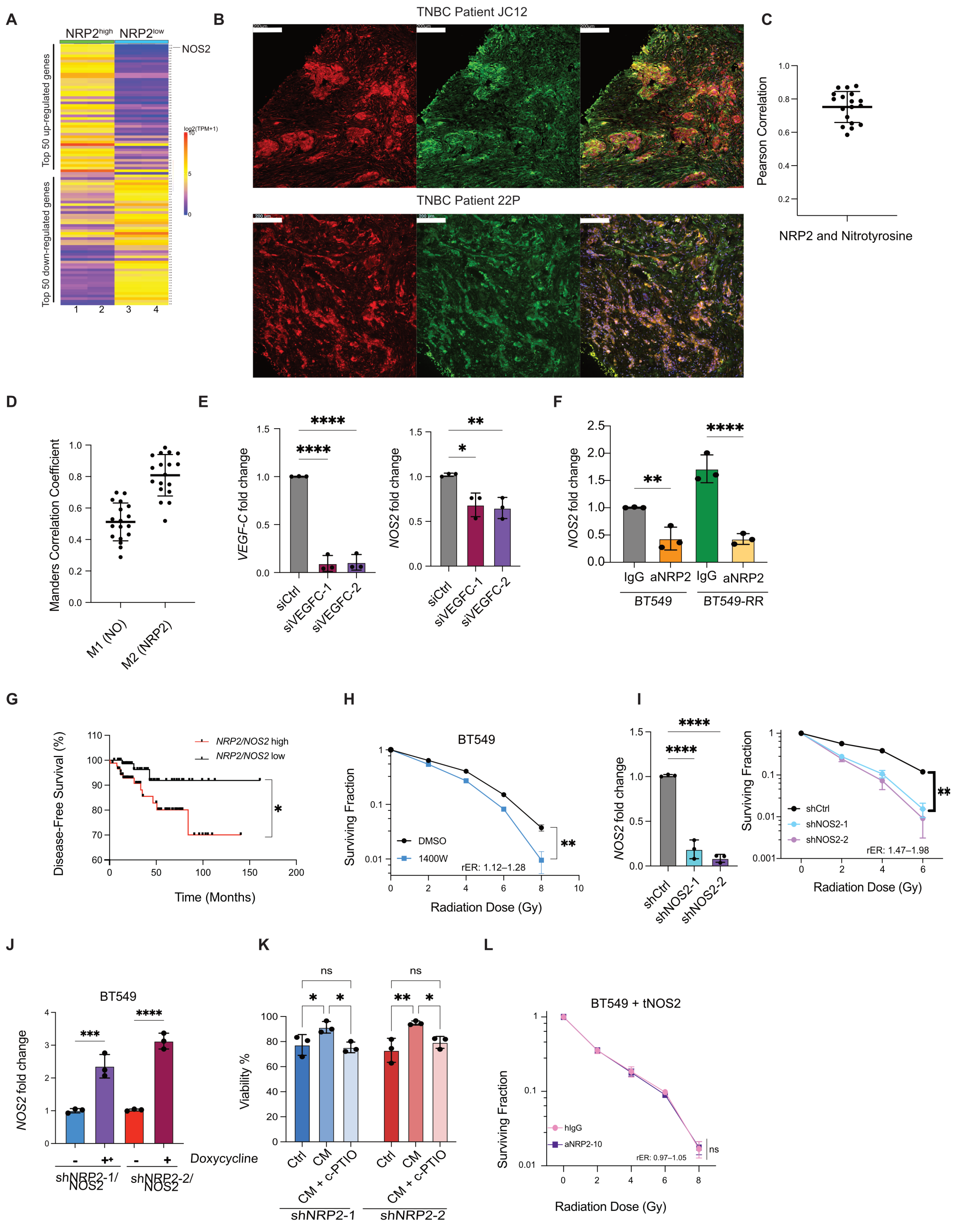

A
NRP2high NRP2low
NOS2
Top 50 up-regulated genes
Top 50 down-regulated genes
log2(TPM+1)
1 2 3 4
B
TNBC Patient JC12
TNBC Patient 22P
C
Pearson Correlation
NRP2 and Nitrotyrosine
D
Manders Correlation Coefficient
M1 (NO)
M2 (NRP2)
E
VEGF-C fold change
NOS2 fold change
siCtrl
siVEGFC-1
siVEGFC-2
F
NOS2 fold change
IgG aNRP2 IgG aNRP2
BT549
BT549-RR
G
Disease-Free Survival (%)
NRP2/NOS2 high
NRP2/NOS2 low
Time (Months)
H
BT549
Surviving Fraction
DMSO
1400W
rER: 1.12–1.28
Radiation Dose (Gy)
I
NOS2 fold change
shCtrl
shNOS2-1
shNOS2-2
Surviving Fraction
rER: 1.47–1.98
Radiation Dose (Gy)
J
BT549
NOS2 fold change
Doxycycline
shNRP2-1/ NOS2
shNRP2-2/ NOS2
K
Viability %
Ctrl
CM
CM + c-PTIO
shNRP2-1
shNRP2-2
L
BT549 + tNOS2
Surviving Fraction
hIgG
aNRP2-10
rER: 0.97–1.05
Radiation Dose (Gy)

Supplemental Figure 2: Nitric oxide production from VEGF/NRP2 and its association with radiation resistance (A) RNA-seq data showing the transcriptomic differences between $NRP2^{high}$ and $NRP2^{low}$ populations of BT549 cells, with 2 samples in each group. **(B)** Representative IHC images of a TNBC patient tumor immunostained with antibodies against NRP2, nitrotyrosine, and DAPI. Scale bar, 200µm. (**C**) The Pearson correlation coefficient of NRP2 and NOS2 was evaluated using four randomly chosen areas from four different TNBC patient tumors. (**D**) The Mander's correlation coefficient of NRP2 and NOS2 was evaluated using four randomly chosen areas from four different TNBC patient tumors. **(E)** *VEGF-C* and *NOS2* mRNA expression in BT549 parental cells transfected with siRNAs targeting VEGF-A (siVEGFA-1, siVEGFA-2) compared to siCtrl as quantified by qPCR at 3 days after transfection (n=3). **(F)** NOS2 transcript levels in parental BT549 and BT549-RR cells given IgG or aNRP2-10 (10µg/mL) for 24 hours (n=3). **(G)** Kaplan-Meir graph of the disease-free survival of breast cancer patients given radiation when segregated by high NRP2 and NOS2 expression vs low NRP2 and NOS2 expression. The dataset was extracted using RNA-seq and clinical data collated from TCGA. Gehan-Breslow-Wilcoxon test with *$P<0.05$ **(H)** Clonogenic assay of BT549 cells treated with 1400W (50µM) or DMSO and irradiated (0-8 Gy; n=2, representative image). **(I)** BT549 cells transfected with shCtrl or shRNAs targeting NOS2 (shNOS2-1, shNOS2-2) and validated by qPCR with its clonogenic assay after being irradiated (0-6 Gy; n=2, representative image). **(J)** The *NOS2* expression based on qPCR of NRP2 knockdown BT549 cells expressing tNOS2 with and without doxycycline stimulation (n=3). **(K)** Cell viability, assessed by Cell-Titer Glo luminescence assay, of NRP2 knockdown cells for the three conditions described in Fig 2E 24h after a radiation dose of 4Gy (n=3). **(L)** Clonogenic assay of BT549 cells transfected with tNOS2 when given antibody treatment (IgG or aNRP2-10) and irradiated (0-8 Gy; n=2, representative image). Data are

presented as the mean ± SD in **C**-**F** and **H**-**L.** Statistical analysis was performed using 1-way ANOVA multiple comparisons in **E**, **F, I** (left), **J**, and **K**, or 2-way ANOVA multiple comparisons in **H, I** (right) and **L**.

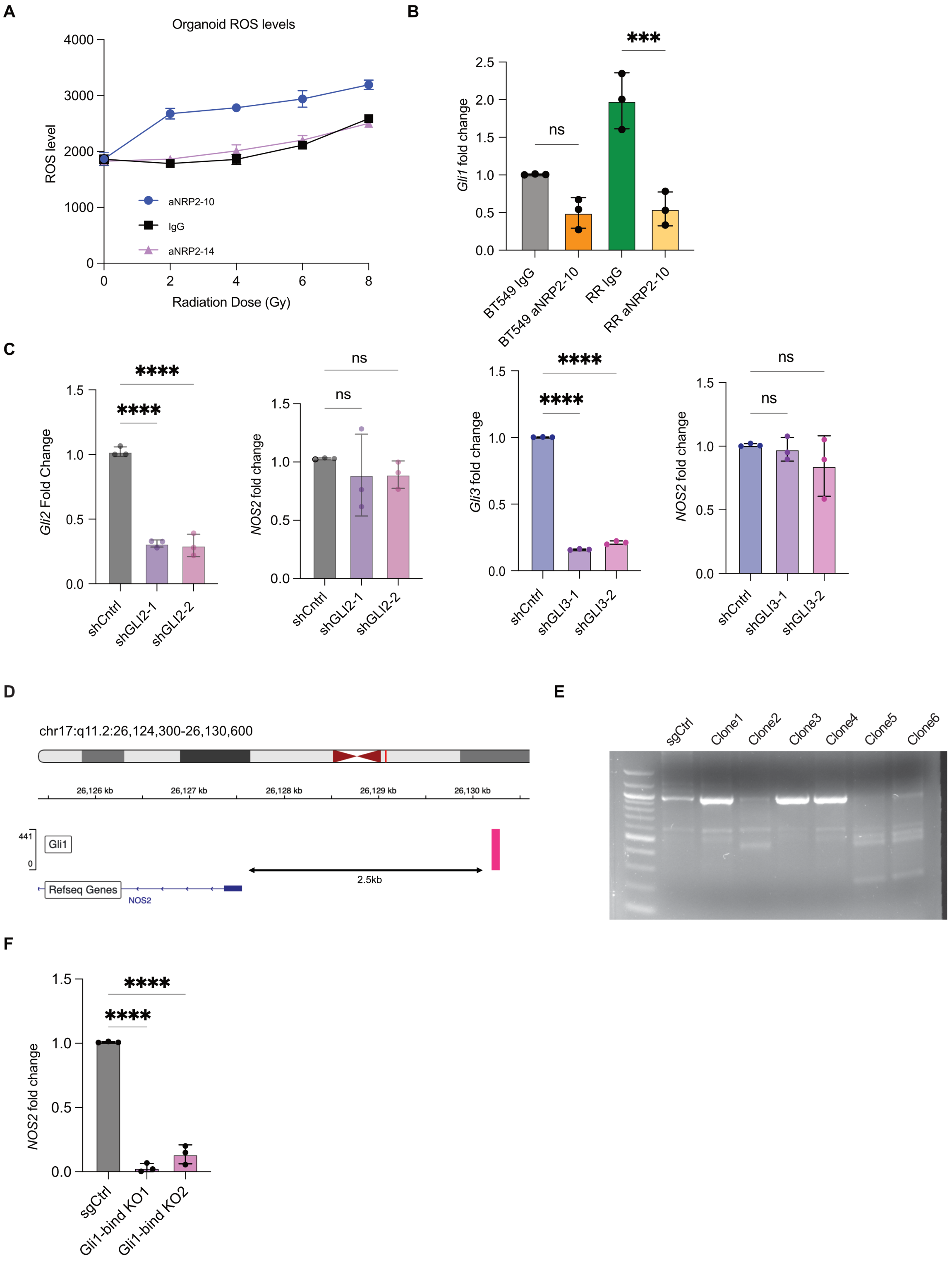
A
Organoid ROS levels
ROS level
4000
3000
2000
1000
0
0
2
4
6
8
Radiation Dose (Gy)
aNRP2-10
IgG
aNRP2-14
B
Gli1 fold change
2.5
2.0
1.5
1.0
0.5
0.0
ns
***
BT549 IgG
BT549 aNRP2-10
RR IgG
RR aNRP2-10
C
Gli2 Fold Change
****
****
shCntrl
shGLI2-1
shGLI2-2
NOS2 fold change
ns
ns
shCntrl
shGLI2-1
shGLI2-2
Gli3 fold change
****
****
shCntrl
shGLI3-1
shGLI3-2
NOS2 fold change
ns
ns
shCntrl
shGLI3-1
shGLI3-2
1.5
1.0
0.5
0.0
D
chr17:q11.2:26,124,300-26,130,600
26,126 kb
26,127 kb
26,128 kb
26,129 kb
26,130 kb
441
0
Gli1
Refseq Genes
NOS2
2.5kb
E
sgCtrl
Clone1
Clone2
Clone3
Clone4
Clone5
Clone6
F
NOS2 fold change
1.5
1.0
0.5
0.0
****
****
sgCtrl
Gli1-bind KO1
Gli1-bind KO2

Supplemental Figure 3: The role of Gli1 on NOS2 expression (A) Quantification of radiation-induced ROS levels in IgG, aNRP2-10, and aNRP2-14 treated organoid (T9441) at varying radiation doses (0-8Gy) at 1hr after radiation (n=3). **(B)** Gli1 mRNA expression based on qPCR of parental BT549 and BT549-RR cells given aNRP2-10 or IgG treatment for 24 hours (n=3). **(C)** The expression of NOS2, Gli2, and Gli3 when knocking down Gli2 or Gli3 based on RT-qPCR (n=3). **(D)** ChIP-seq data of Gli1 from GSE100936 identifies a binding site 2.5kb upstream of the *NOS2* promoter. **(E)** Genomic DNA from clones were isolated, amplified with PCR, and processed in an agarose gel to determine the efficiency of deleting the Gli1 binding region of the NOS2 promoter in BT549 cells. The wildtype band is predicted to be at 1000bp and the deleted region results in band of 450bp. Clone 2 (Gli-bind KO1) and Clone 5 (Gli-bind KO2) were selected as Gli-binding region knockouts described in Figure 4I and 4J. (**F**) *NOS2* mRNA expression of CRISPR generated mutations of the Gli1 binding site (Gli1-bind KO1 and KO2) compared to control (n = 3). Data are presented as the mean ± SD in **A-C,** and **F**. Statistical analysis was performed using 1-way ANOVA multiple comparisons in **B**, **C**, and **F**, or 2-way ANOVA multiple comparisons in **A**.

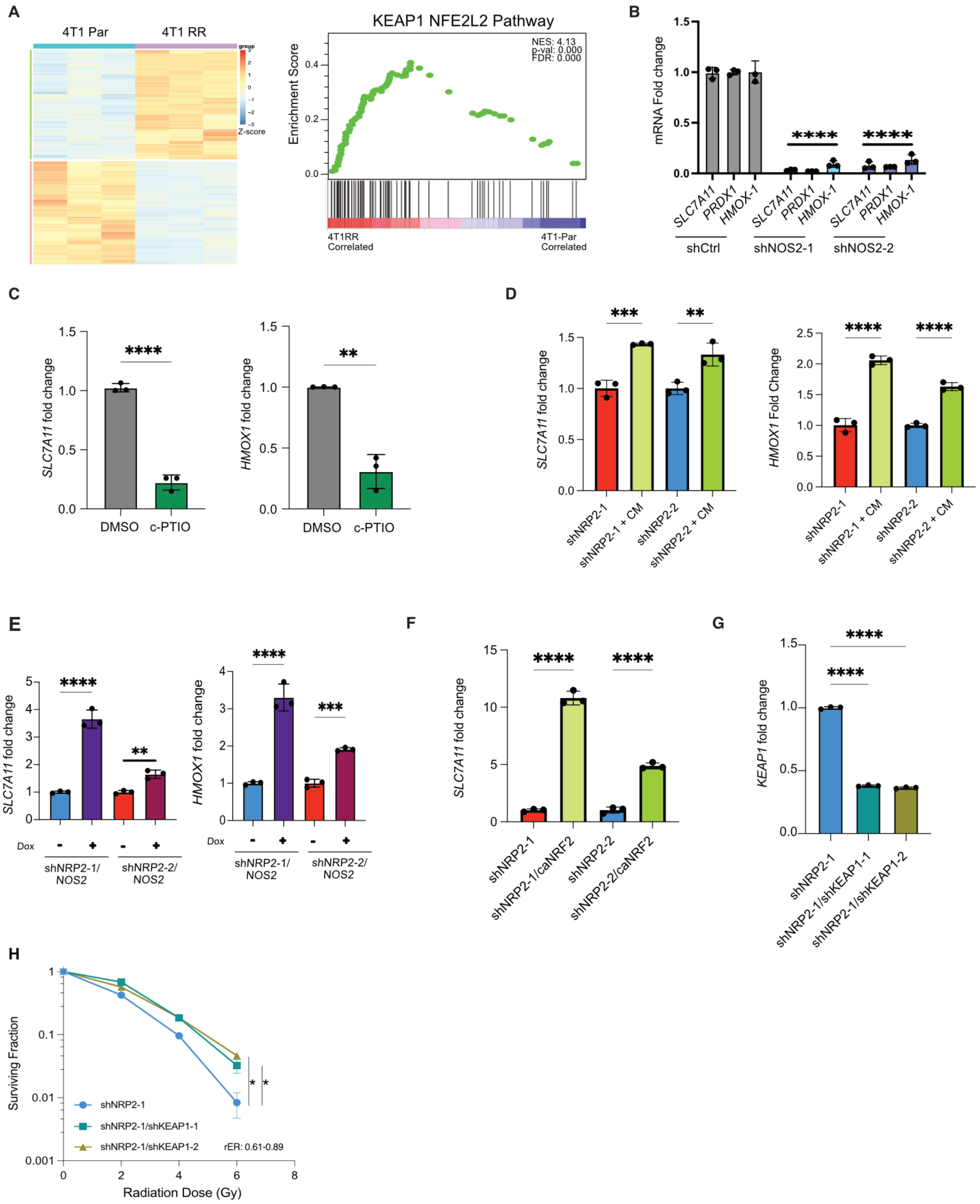

A
4T1 Par
4T1 RR
group
Z-score
KEAP1 NFE2L2 Pathway
NES: 4.13
p-val: 0.000
FDR: 0.000
Enrichment Score
4T1RR Correlated
4T1-Par Correlated
B
mRNA Fold change
SLC7A11
PRDX1
HMOX-1
shCtrl
shNOS2-1
shNOS2-2
****
C
SLC7A11 fold change
HMOX1 fold change
DMSO
c-PTIO
****
**
D
SLC7A11 fold change
HMOX1 Fold Change
shNRP2-1
shNRP2-1 + CM
shNRP2-2
shNRP2-2 + CM
***
**
****
E
SLC7A11 fold change
HMOX1 fold change
Dox
shNRP2-1/ NOS2
shNRP2-2/ NOS2
****
**
***
F
SLC7A11 fold change
shNRP2-1
shNRP2-1/caNRF2
shNRP2-2
shNRP2-2/caNRF2
****
G
KEAP1 fold change
shNRP2-1
shNRP2-1/shKEAP1-1
shNRP2-1/shKEAP1-2
****
H
Surviving Fraction
Radiation Dose (Gy)
shNRP2-1
shNRP2-1/shKEAP1-1
shNRP2-1/shKEAP1-2
rER: 0.61-0.89

Supplemental Figure 4. Nitric oxide drives NFE2L2 Activation and radiation resistance (A) RNA-seq data of 4T1 parental vs 4T1-RR with the corresponding enrichment plot for the NFE2L2/KEAP1 pathway. **(B)** *PRDX1*, *HMOX1* and *SLC7A11* mRNA expression in control and shNOS2 BT549 cells (n=3). (**C**) *HMOX1* and *SLC7A11* mRNA expression in BT549 cells treated with DMSO or c-PTIO (50uM) for 24 hours (n=3). (**D**) *HMOX1* and *SLC7A11* mRNA expression in NRP2 depleted cells given normal medium or NRP2^{high} conditioned medium (n=3). (**E**) *HMOX1* and *SLC7A11* mRNA expression in NRP2 knock down cells transfected with tNOS2 with and without doxycycline stimulation (n=3). (**F**) SLC7A11 mRNA expression in NRP2 depleted cells transfected with caNFE2L2 or empty vector (n=3). (**G**) Validation of KEAP1 knockdown in shNRP2-1 BT549 cells using qPCR (n=3). (**H**) Clonogenic assay of BT549 NRP2 knockdown cells that had been stably transfected with shRNAs targeting KEAP1 and then irradiated (0-6Gy; n=2, representative image). Data are presented as the mean ± SD in **B-G.** Statistical analysis was performed using 2-tailed Student's t test in **C**, 1-way ANOVA multiple comparisons in **D-G,** or 2-way ANOVA multiple comparisons in **H**.

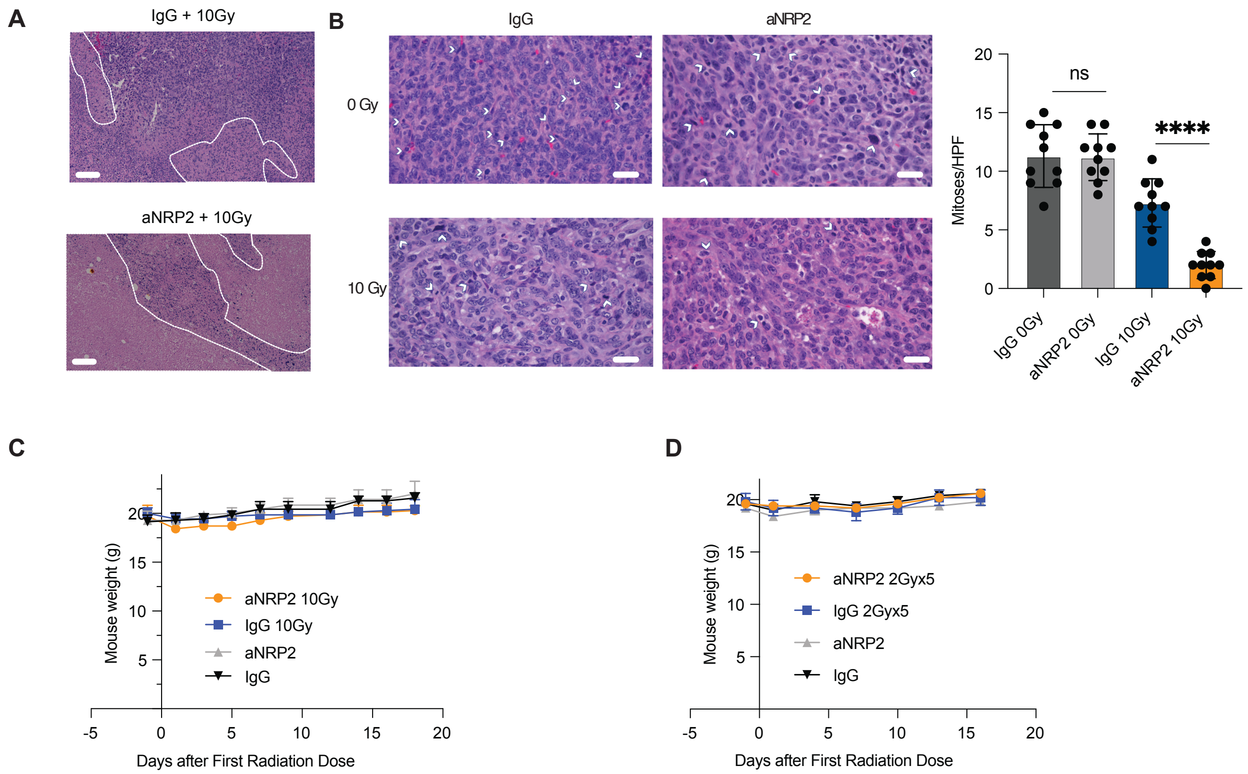

Supplemental Figure 5. Single dose and conventional fractionation radiation effects on 4T1 tumors in vivo (A) Representative images of necrosis in the tumors treated with irradiation in combination with either IgG or aNRP2-28. Necrotic regions are outlined with a white dashed line. Scale bar, 100µm. **(B)** Representative images of mitotic cells found in each 400x field from all the four treatment groups. A total of 10 fields from each group was taken and used for analysis of proliferation. Scale bar, 25µm. (**C**) Mouse weights for the four groups of 4T1 mammary tumors given antibody and/or 10Gy single dose irradiation. (**D**) Mouse weights for the four groups of 4T1 mammary tumors given antibody and/or conventional fractionation radiation of 2gyx5. Data are presented as the mean ± SD in **B**-**D.** Statistical analysis was performed using 1-way ANOVA multiple comparisons in **B**, or 2-way ANOVA multiple comparisons in **C** and **D**.

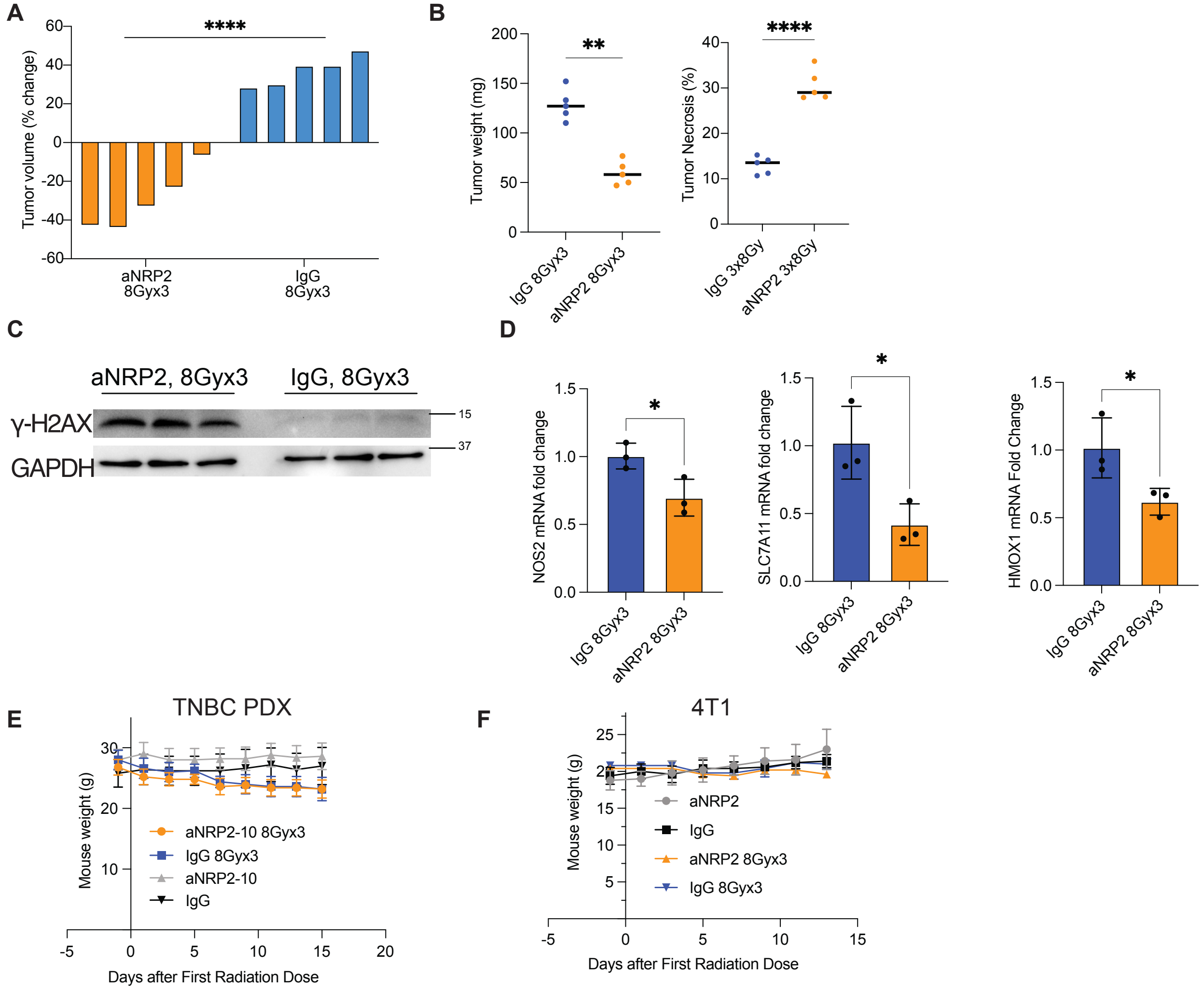

Supplemental Figure 6: Hypofractionated radiation with aNRP2-10 treatment induces tumor regression (A) 4T1 cells (5 x 10^5) were injected into the mammary fat pads of BALB/c mice. Once the tumor volume reached approximately $100mm^3$, the mice were divided into 4 groups of 5 mice each (mouse IgG 0Gy, mouse IgG 8Gy/day for 3 days, aNRP2 0Gy, aNRP2 8Gy/day for 3 days). The mice were given i.p. injections of the specified antibody (25mg/kg) every 48 hours starting 1 day prior to irradiation. Tumors were extracted on day 13. The waterfall plot shows the percent change in growth of the tumor from day -1 to day 13 for each individual mouse. **(B)** The tumor weights from the radiation treated groups. Percent tumor necrosis based on pathological analysis of H&E section of the tumors from the radiation treated groups. **(C)** Immunoblot of g-H2AX from three mice in each of the radiation treated groups **(D)** NOS2, SLC7A11, and HMOX1 mRNA expression in tumors from three mice in each of the radiation treated groups. (**E**) Mouse weights for the four groups of TNBC PDX mammary tumors given antibody and/or hypofractionation radiation of 8Gyx3**.** (**F**) Mouse weights for the four groups of 4T1 mammary tumors given antibody and/or hypofractionation radiation of 8Gyx3**.** Data are presented as the mean ± SD in **B**, and D-F**.** Statistical analysis was performed using 2-tailed Student's t test in **B** and **D**, or 2-way ANOVA multiple comparisons in **E** and **F**.

Supplemental Table 1: Identified Surface Proteins with increased expression after Radiation in TNBC (FC>1.25) and no change in Normal Mammary Epithelial cells (FC<1)

UniProt gene	Fold Change 9Gy (Normal)	Fold Change 23Gy (Normal)	Fold Change 9Gy (TNBC)	Fold Change 23Gy (TNBC)
ADRB2	0.87	0.83	1.87	2.46
BMPR1A	0.75	0.64	1.39	1.53
CD40	0.87	0.93	1.45	1.40
EPHA2	0.94	0.90	1.31	1.47
EPHB1	1.00	0.87	1.35	2.40
FCAMR	0.96	0.69	1.51	1.62
ITGB8	0.98	1.00	1.56	1.28
KIT	1.00	1.00	1.82	1.80
KLRB1	0.58	0.98	1.29	1.30
MRGPRX3	0.94	0.96	1.44	1.54
NPR3	0.65	0.82	1.58	1.46
NRP2	0.90	0.90	1.28	1.52
NTRK1	0.86	0.70	1.34	1.32
OLR1	0.94	0.84	1.32	1.30
OR10J5	0.94	0.86	1.40	1.28
OR12D3	0.92	0.81	1.59	1.53
PTGFR	1.00	1.00	1.30	1.46
PTPRO	0.76	0.62	1.27	2.14
RYK	0.98	0.84	1.38	1.46
SDC1	0.99	0.95	1.35	2.36
TEK	1.00	0.96	1.32	1.63
TNFRSF18	0.84	0.93	1.69	1.79

Supplemental Table 2: Normal Mammary Gland Histology after Treatment

Mouse	Atypia	Apoptosis	Inflammation	Radiation Dose	Antibody Treatment
5169	+ (mild)	+	-	10Gy	IgG
5158	+ (mild)	+	-	10Gy	IgG
5164	+ (mild)	+	-	10Gy	IgG
5163	+ (mild)	+	+ (rare lymphocyte)	10Gy	aNRP2-28
5167	+ (mild)	+	-	10Gy	aNRP2-28
5165	+ (mild)	+	-	10Gy	aNRP2-28
5655	-	+	+ (rare lymphocyte)	2Gyx5	aNRP2-28
5664	-	+	-	2Gyx5	aNRP2-28
5661	-	+	+ (rare lymphocyte)	2Gyx5	aNRP2-28
5558	-	+	-	2Gyx5	aNRP2-28
5654	-	+	+ (rare lymphocyte)	2Gyx5	aNRP2-28
5662	-	+	+ (rare lymphocyte)	2Gyx5	aNRP2-28
5666	-	+	+ (rare lymphocyte)	2Gyx5	IgG
5665	-	+	+ (rare lymphocyte)	2Gyx5	IgG
5656	-	+	-	2Gyx5	IgG

5651	-	+	-	2Gyx5	IgG
5647	-	+	-	2Gyx5	IgG
5652	-	+	-	2Gyx5	IgG

Supplemental Table 3: Primers

Gene of Interest	Forward Primer	Reverse Primer
hNRP2	GGATGGCATTCCACATGTTG	ACCAFFTAGTAACFCFCAGAG
mNRP2	TTCTCTGCAAGGGACACAGAC	TAAGCCATCCTCTTCTGCCT
hNOS2	TCGGCAGAATCTACAAAGTGC	TGGCCATCCTCACAGGAG
mNOS2	GGAGTGACGGCAAACATGACT	TCGATGCACAACTGGGTGAAC
hSLC7A11	TCTCCAAAGGAGGTTACCTGC	AGACTCCCCTCAGTAAAGTGA
mSLC7A11	GGCACCGTCATCGGATCAG	CTCCACAGGCAGACCAGAAAA
hHMOX1	AAGACTGCGTTCCTGCTCAAC	AAAGCCCTACAGCAACTGTCG
mHMOX1	GATAGAGCGCAACAAGCAGAA	CAGTGAGGCCCATACCAGAAG
hPRDX1	CATTCCTTTGGTATCAGACCCG	CCCTGAACGAGATGCCTTCAT
mPRDX1	AATGCAAAAATTGGGTATCCTGC	CCGTGGGACACACAAAAGTAAA
hKEAP1	GTGTCCATTGAGGGTATCCACC	GCTCAGCGAAGTTGGCGAT
hVEGF-c	GAGGAGCAGTTACGGTCTGTG	TCCTTTCCTTAGCTGACACTTGT
mGAPDH	AGGTCGGTGTGAACGGATTTG	GGGGTTCGTTGATGGCAACA
hGAPDH	GGAGCGAGATCCCTCCAAAAT	GGCTGTTGTCATACTTCTCAT
hGli1	AGCGTGAGCCTGAATTCTGTG	CAGCATGTACTGGGCTTTGAA
mGli1	CTCGACCTGCAAACCGTAATC	TCCTAAAGAAGGGCTCATGGTA
