# Supplementary material for: Neuropilin-2–expressing breast cancer cells mitigate radiation-induced oxidative stress through nitric oxide signaling
Source: J Clin Invest. 2024 Oct 1;134(22):e181368. doi: 10.1172/JCI181368 (PMC11563673; doi:10.1172/JCI181368)

Unedited blot Figure 1C BT549 NRP2

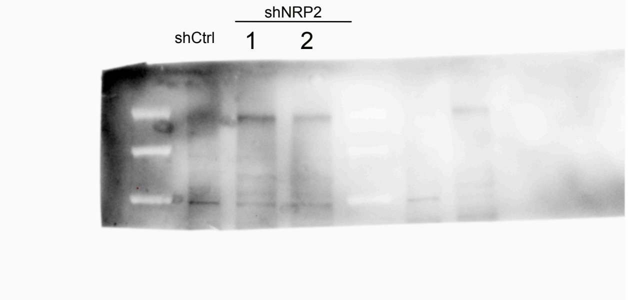

Unedited blot Figure 1C BT549 gapdh

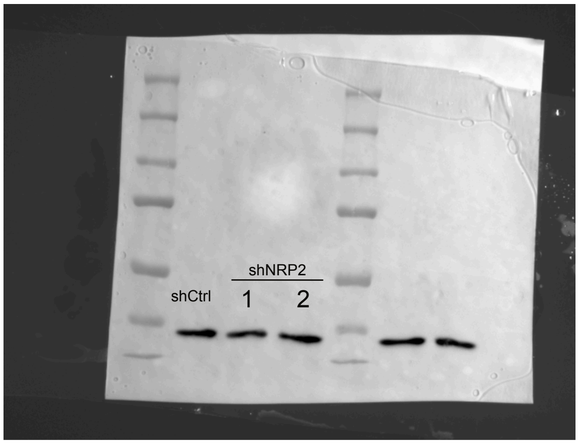

Unedited blot Figure 1C 4T1 nrp2

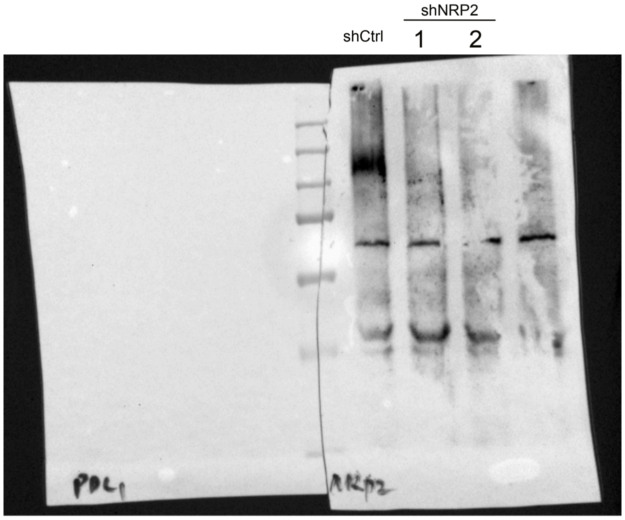

Unedited Blot Figure 1C 4T1 Gapdh

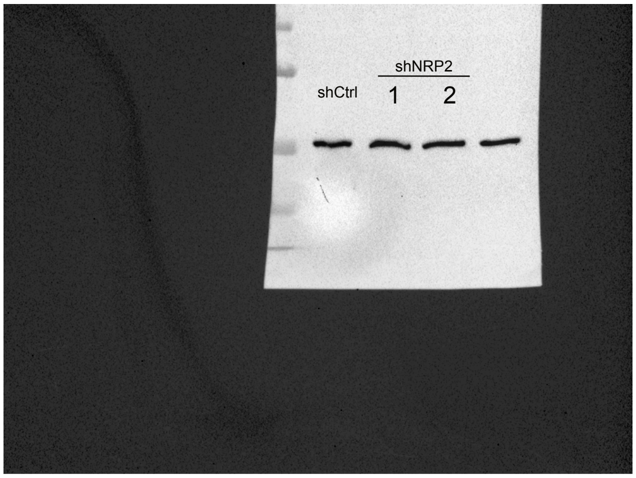

Unedited blot Figure 2C BT549 NOS2

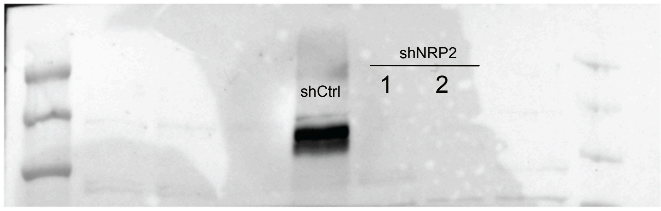

Unedited blot Figure 2C BT549 gapdh

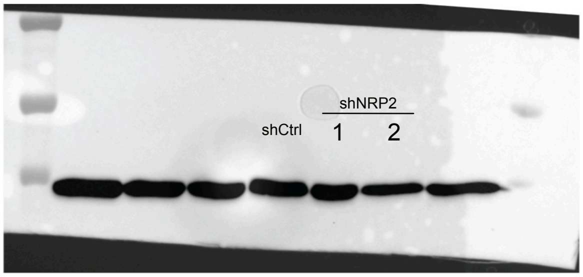

Unedited blot Figure 2C 4T1 NOS2

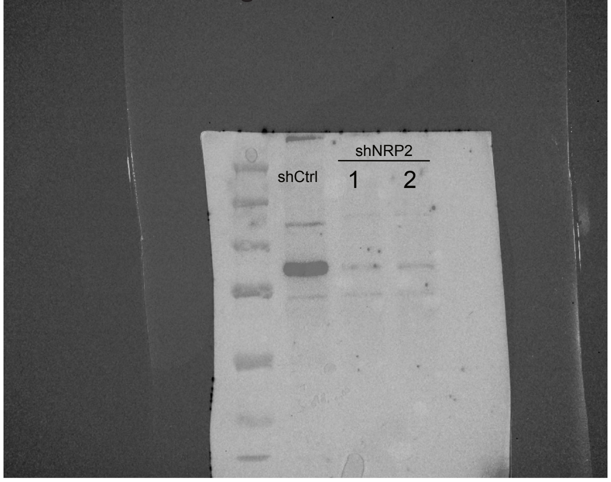

Unedited blot Figure 2C 4T1 GAPDH

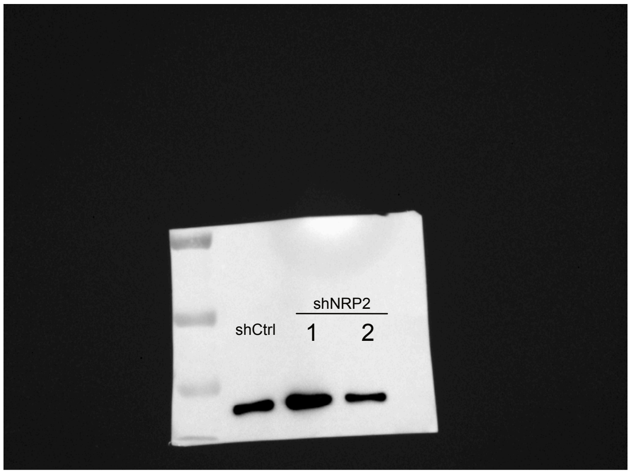

Unedited blot Figure 2E shnrp2-1 nitrotyrosine

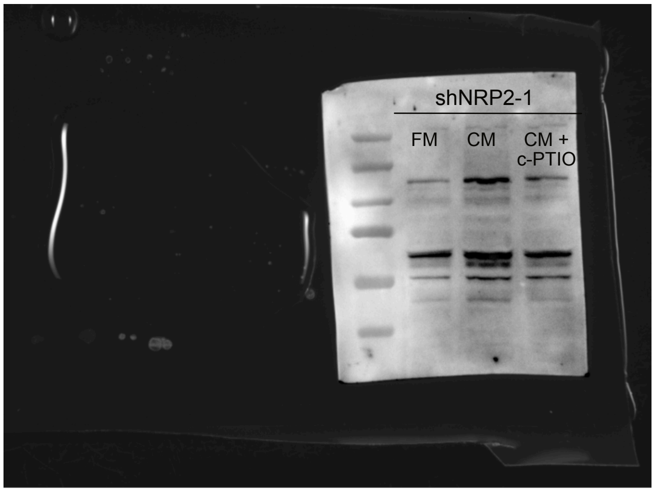

Unedited blot Figure 2E shnrp2-1 gapdh

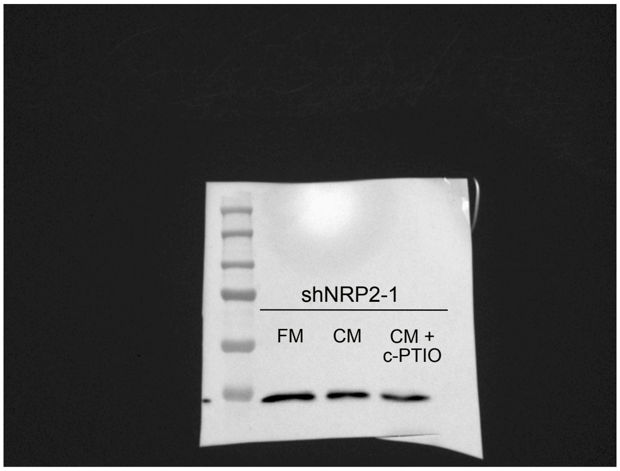

Unedited blot Figure 2E shnrp2-2 nitrotyrosine

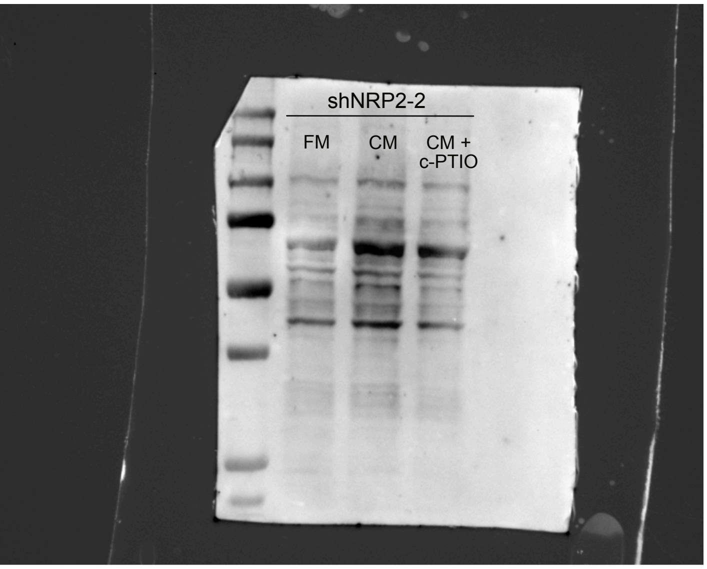

Unedited blot Figure 2E shnrp2-2 tubulin

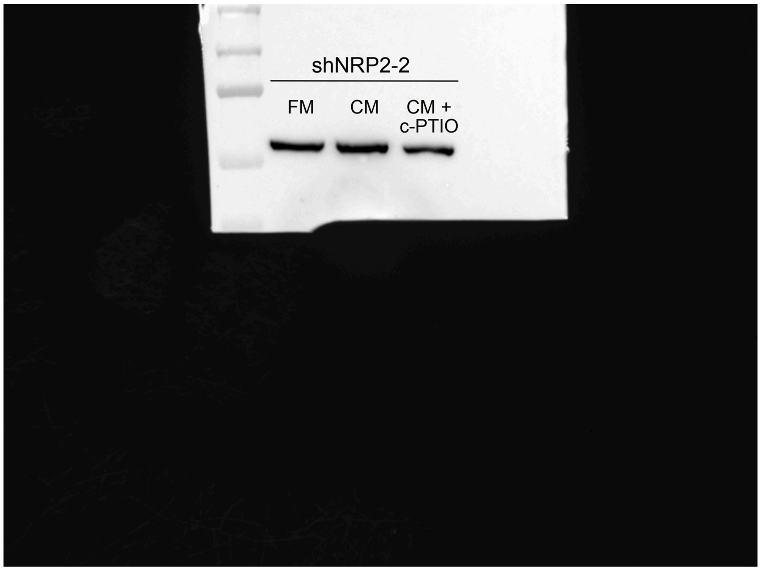

Unedited blot Figure 4F Gli1

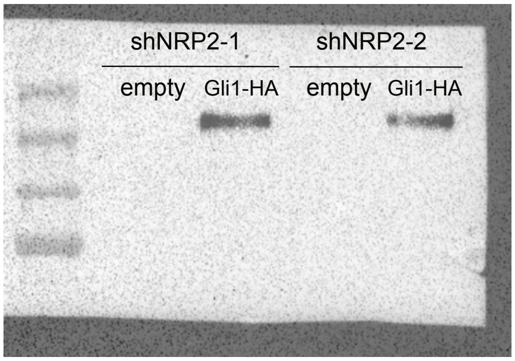

Unedited Blot Figure 4F nos2

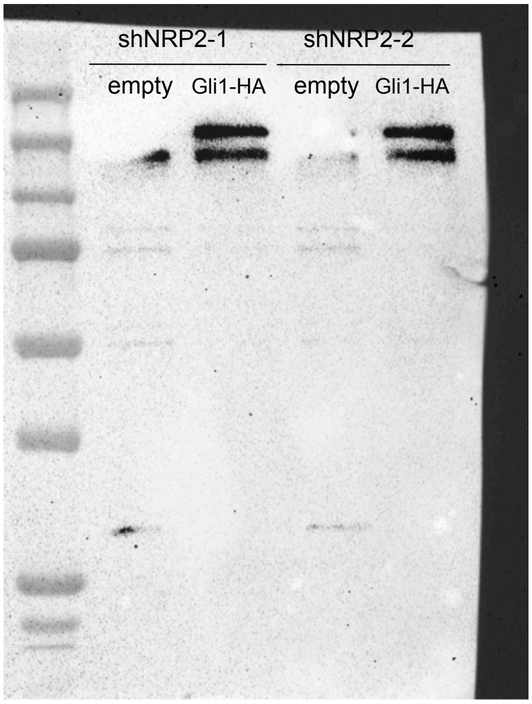

Figure 4E gapdh

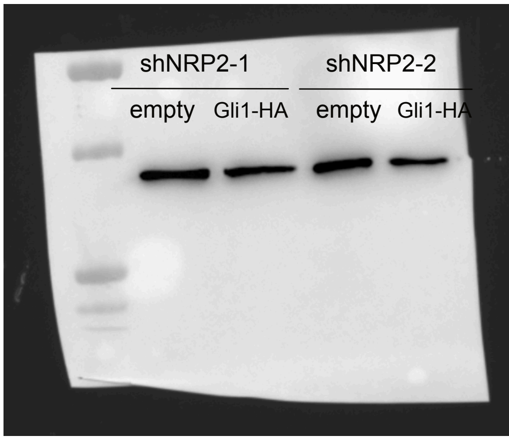

Unedited blot Figure 4H nos2

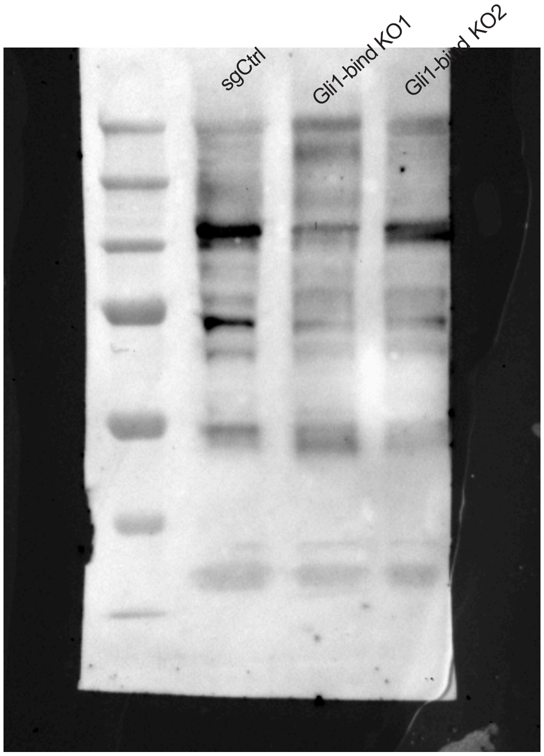

Unedited blot Figure 4H tubulin

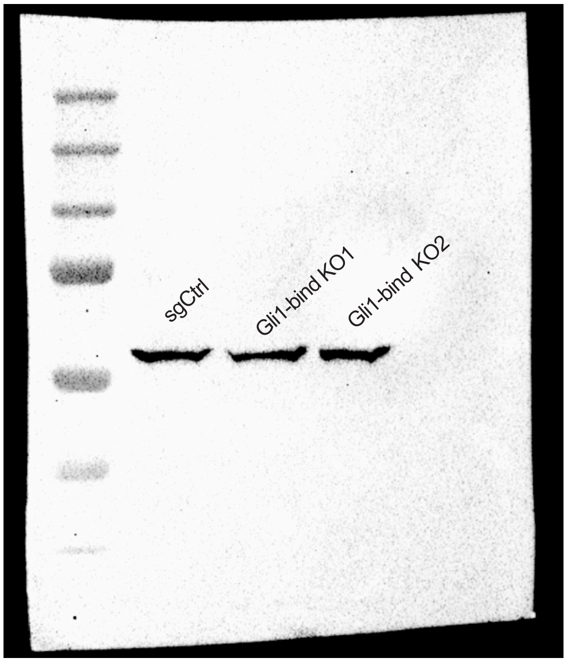

Unedited blot Figure 5F keap1 streptavidin

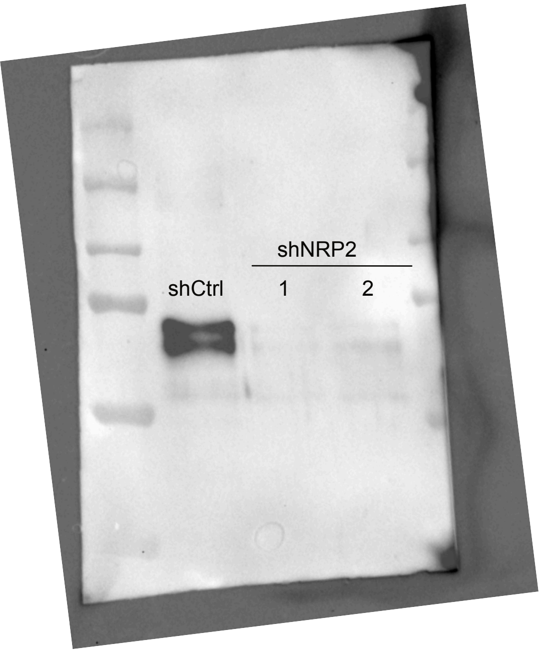

Figure 5F keap1 input

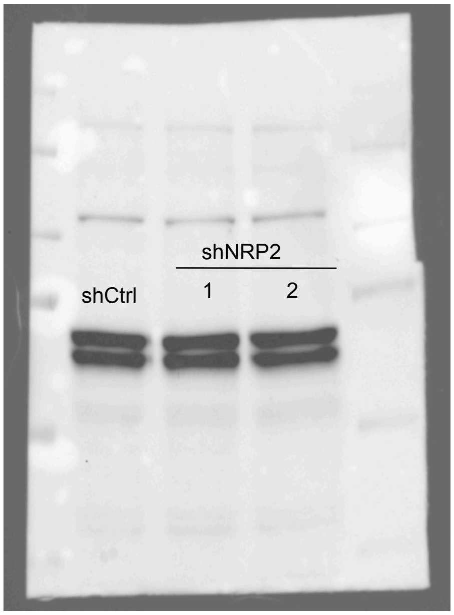

Unedited blot for Figure 5G keap1 anti-TMT

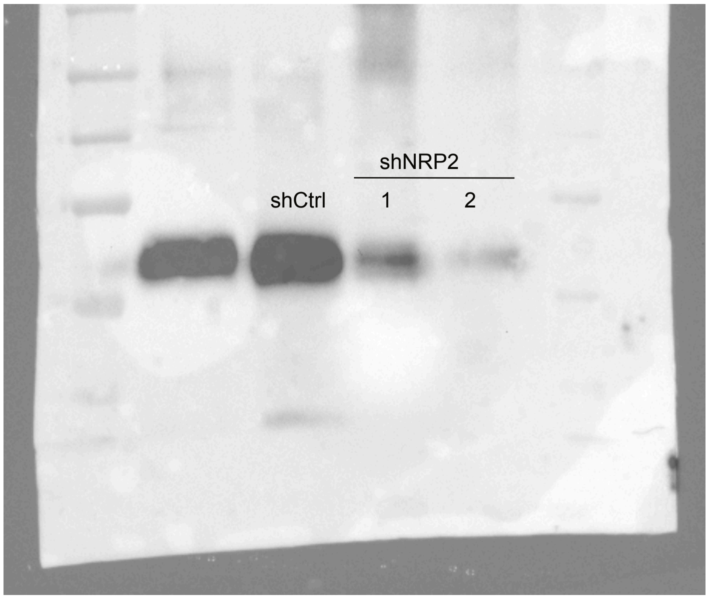

Unedited blot for Figure 5G keap1 input

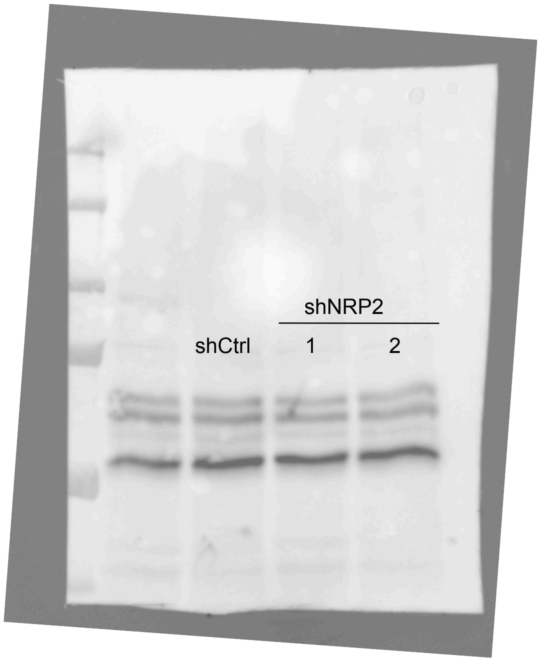

Unedited blot for Figure 6C gammah2ax

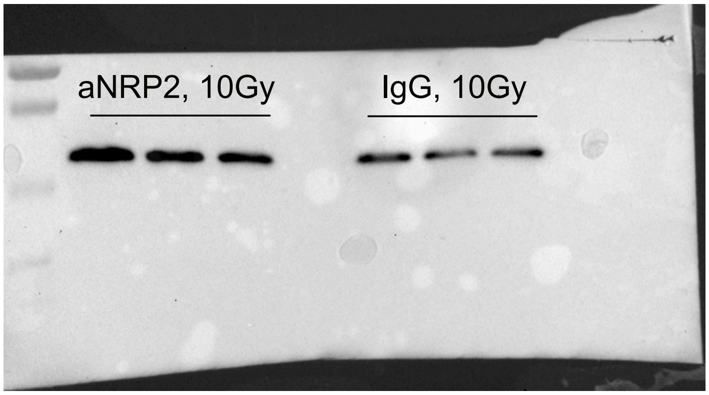

Unedited blot for Figure 6C gapdh

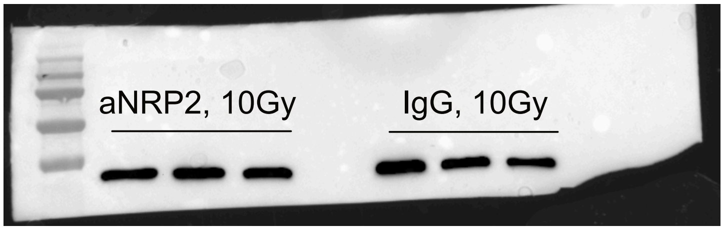

Unedited blot for Figure 6F nos2

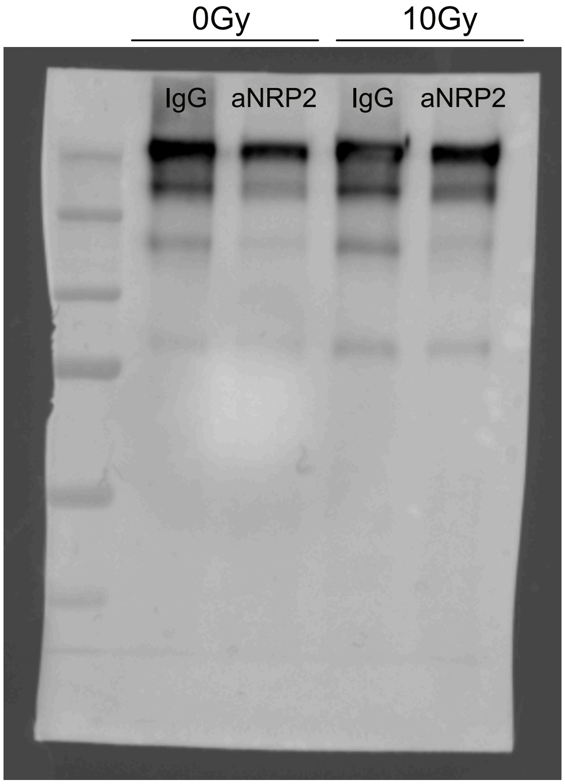

Unedited blot for Figure 6F tubulin

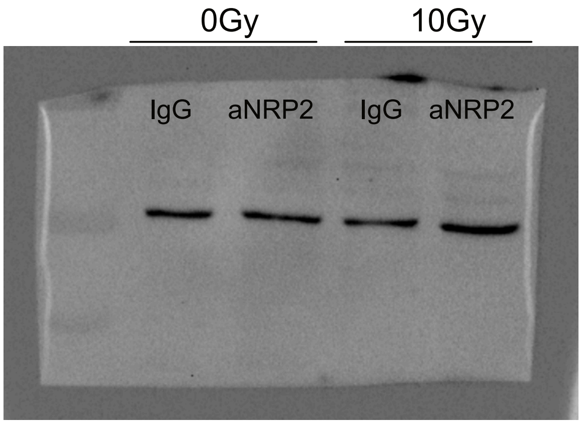

Unedited blot for Figure 7C gammah2ax

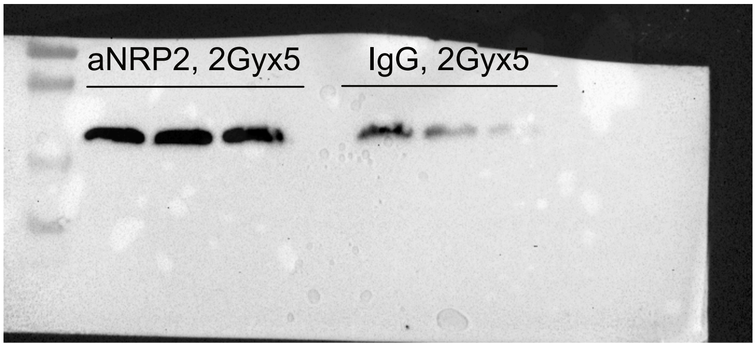

Unedited blot for Figure 7C gapdh

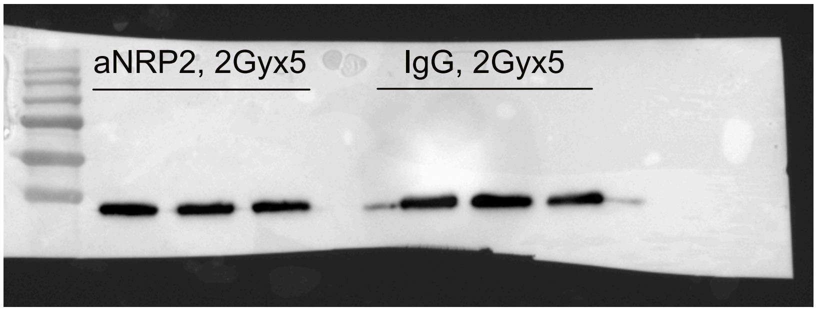

Unedited blot for Figure 7D NOS2

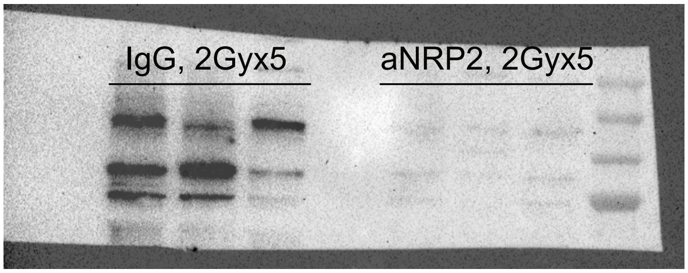

Unedited blot for Figure 7D gapdh

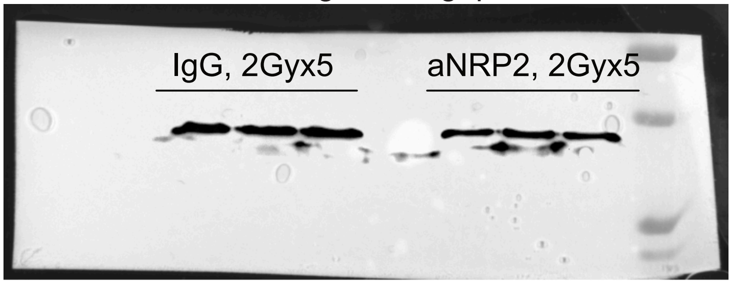

Unedited blot for Figure 8D gammah2ax

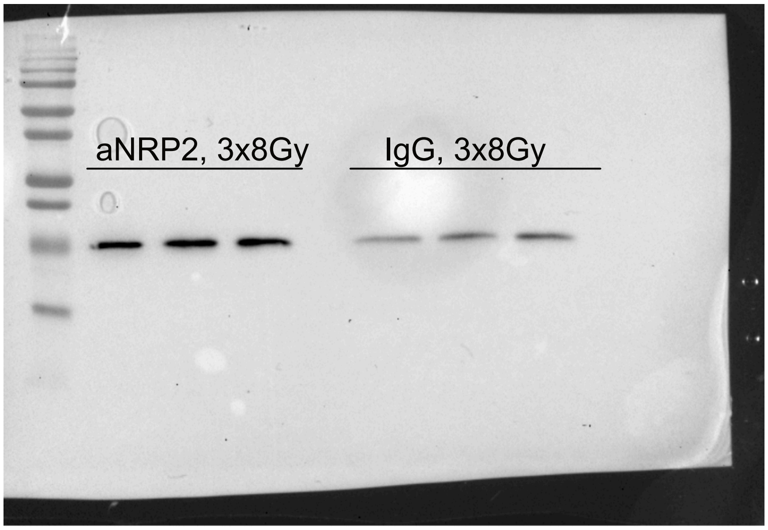

Unedited blot for figure 8D gapdh

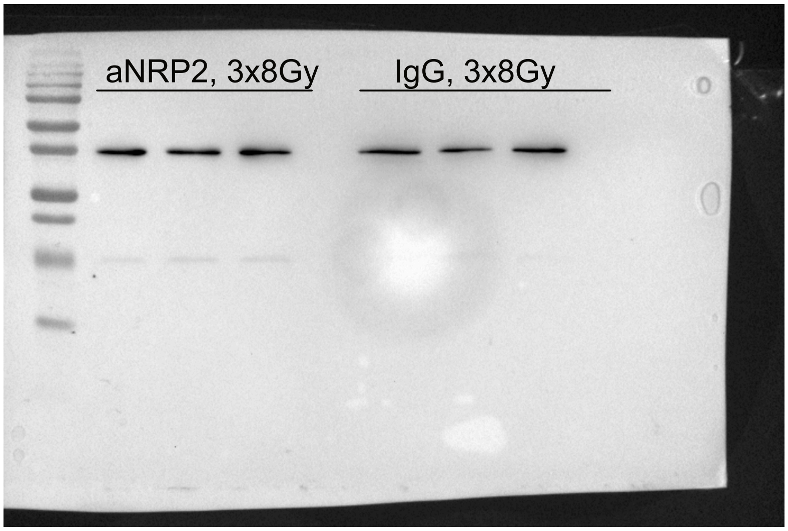

Unedited gel for Figure S3E CRISPR Gli1 binding site

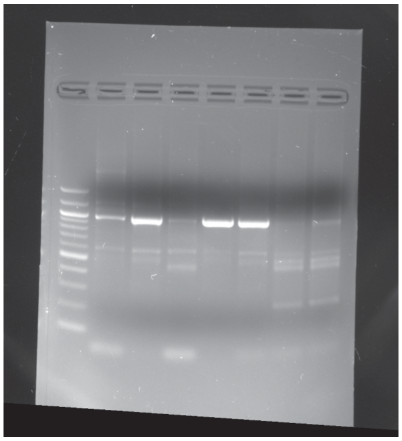

Unedited blot for Figure S6c gammah2ax

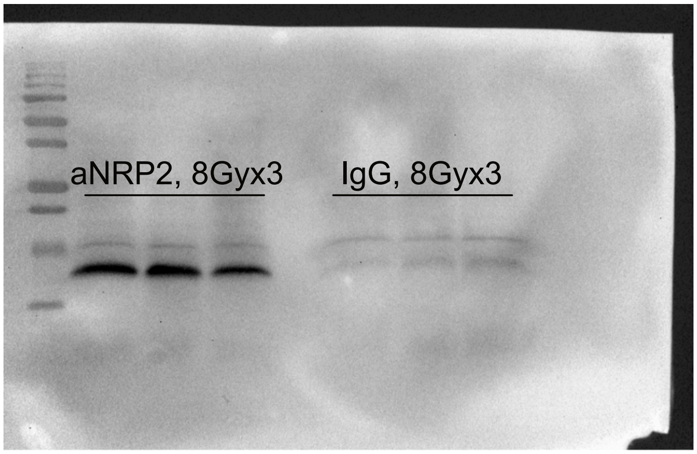

Unedited blot for Figure S6c gapdh

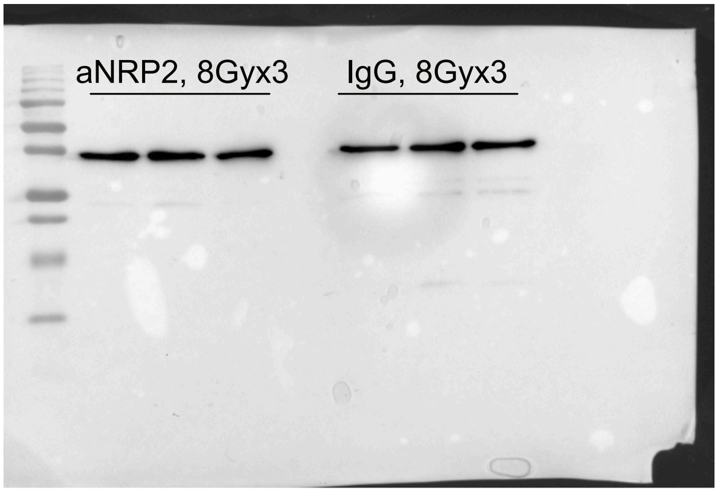

Supplement: Unedited blot and gel images [file jci-134-181368-s010.pdf]
